# Electrophysiology and Morphology of Human Cortical Supragranular Pyramidal Cells in a Wide Age Range

**DOI:** 10.1101/2024.06.13.598792

**Published:** 2024-06-13

**Authors:** Pál Barzó, Ildikó Szöts, Martin Tóth, Éva Adrienn Csajbók, Gábor Molnár, Gábor Tamás

**Affiliations:** 1 Department of Neurosurgery, University of Szeged, Szeged, Hungary; 2 ELKH-SZTE Research Group for Cortical Microcircuits, Department of Physiology, Anatomy and Neuroscience, University of Szeged, Szeged, Hungary

## Abstract

The basic excitatory neurons of the cerebral cortex, the pyramidal cells, are the most important signal integrators for the local circuit. They have quite characteristic morphological and electrophysiological properties that are known to be largely constant with age in the young and adult cortex. However, the brain undergoes several dynamic changes throughout life, such as in the phases of early development and cognitive decline in the aging brain. We set out to search for intrinsic cellular changes in supragranular pyramidal cells across a broad age range: from birth to 85 years of age and we found differences in several biophysical properties between defined age groups. During the first year of life, subthreshold and suprathreshold electrophysiological properties changed in a way that shows that pyramidal cells become less excitable with maturation, but also become temporarily more precise. According to our findings, the morphological features of the three-dimensional reconstructions from different life stages showed consistent morphological properties and systematic dendritic spine analysis of an infantile and an old pyramidal cell showed clear significant differences in the distribution of spine shapes. Overall, the changes that occur during development and aging may have lasting effects on the properties of pyramidal cells in the cerebral cortex. Understanding these changes is important to unravel the complex mechanisms underlying brain development, cognition and age-related neurodegenerative diseases.

## Introduction

After birth, the brain undergoes developmental changes for a prolonged time that involve a series of complex and accurately orchestrated processes (Rakic, 2009). The production and migration of neurons is largely completed at the beginning of postnatal development, and then the intrauterine developmental processes continue: thickening of gray and white matter, myelination, synaptogenesis, pruning, and establishment of the basic anatomical architecture for the initial function of the neural pathways. Subsequently, local connections within cortical circuits are fine-tuned, and increasingly complex, longer-term connections are established between circuits (Stiles & Jernigan, 2010). After that, the changes do not end, but continue throughout human life. They are mostly driven by environmental influences and experiences and lead to changes in metabolic activities (Kuzawa et al., 2014), changes in functional connectivity patterns (Kelly et al., 2009) and, with the maturation of white matter (Beck et al., 2021; Yeatman et al., 2014) changes in the speed of long-distance transmission (van Blooijs et al., 2023). The final phase is aging, where it slowly declines with advancing age, leading to a decline in cognitive signal processing functions and often resulting in neurodegenerative diseases (Peters, 2006). The cortical supragranular glutamatergic cell (or pyramidal cell) provides the excitatory synaptic inputs for local inhibitory circuitry and other pyramidal cells by which they create distinct subnetworks (Yoshimura et al., 2005). The development and formation of dendrites (Koenderink & Uylings, 1995; Petanjek et al., 2008) and synapses (Huttenlocher & Dabholkar, 1997) of pyramidal cells in the human cerebral cortex has been documented to some extent by postmortem studies, besides much less is known about their biophysical maturation and electrical properties in the early stages of development and the subsequent change or maintenance in later ages. Numerous studies demonstrated in non-primate animal models that the electrical characteristics of neurons change prominently in the early postnatal stage (Molnár et al., 2020). Changes in the intrinsic membrane properties (Picken Bahrey & Moody, 2003), the input resistance or the kinetics of the elicited action potentials were reported (Elston & Fujita, 2014; Kroon et al., 2019) in connection with maturation of macaque and rodent pyramidal cells. To date, however, no cross-age studies have been conducted on the electrophysiological parameters of human pyramidal neurons. We have studied in detail the postnatal lifetime profile of the physiological and morphological properties of supragranular (layer 2/3) neurons of human pyramidal cells from neurosurgical resections. To this end, we performed whole-cell patch-clamp recordings and 3D anatomical reconstructions of human cortical pyramidal cells from 108 patients aged 1 month to 85 years for comprehensive data analysis to obtain the morphoelectric lifetime profile of supragranular pyramidal cells.

## Results

### Age-dependent differences in intrinsic subthreshold membrane properties

**Figure 1.**
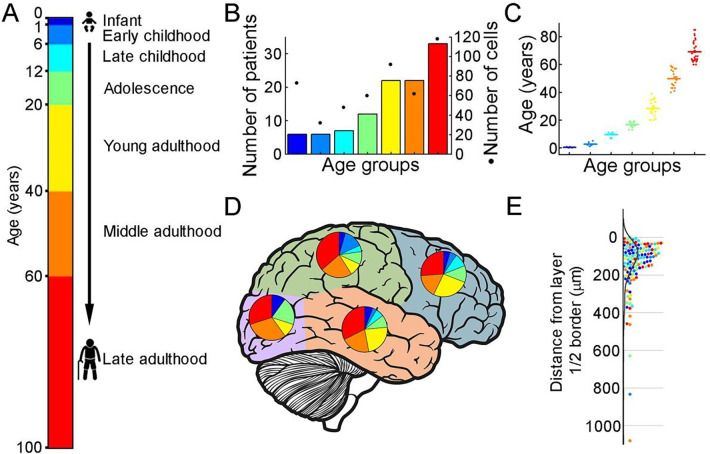
Illustration of the patient data on the samples utilized (A) Illustration of the defined age groups. (B) Number of patients involved in age groups, (n = 6, 6, 7, 12, 22, 22, 33 from infant to late adulthood, respectively). Dots show the number of human layer 2/3 pyramidal cells in our dataset regarding the defined age groups (n = 73, 32, 48, 60, 92, 62, 118 from infant to late adulthood, respectively). (C) Distributions of patient ages within age groups. (D) Brain model indicates the number of surgically removed tissues from the cortical lobes. Colors indicate age groups. (E) The distribution of recovered cell bodies distance from the L1/2 border.

**Figure 2.**
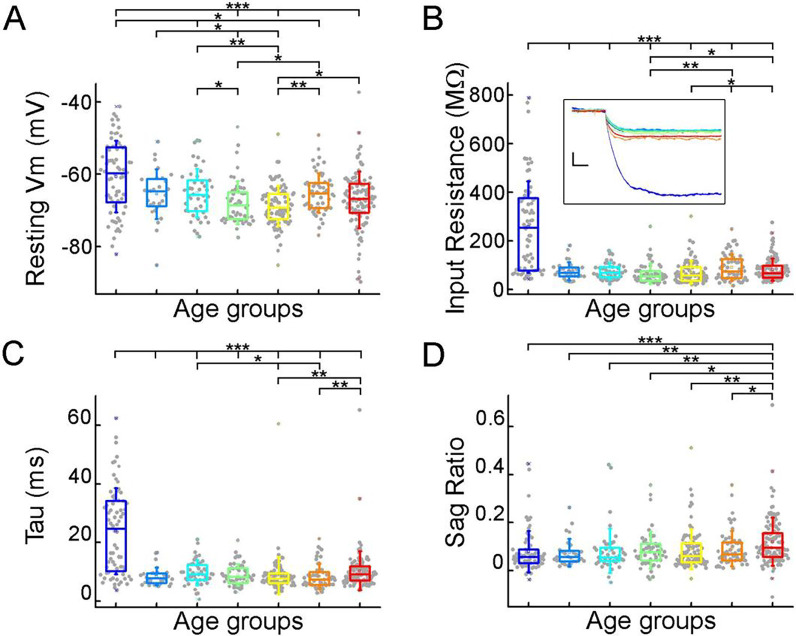
Subthreshold membrane properties vary across life stage (A-D), Boxplots show resting membrane potential (A), input resistance (B), tau (C), and sag ratio (D) distributions in various age groups. (B) Inset shows representative voltage traces from each group. Scale bar: 5 mV; 20 ms. Asterisks indicate significance (Kruskal–Wallis test with post-hoc Dunn test, * P < 0.05, ** P < 0.01, *** P < 0.001).

To extract biophysical properties of excitatory cells of human brain specimens we performed whole cell patch-clamp recordings of pyramidal cells from neurosurgically removed human neocortical tissue sections. The samples were mainly from the frontal and temporal lobes (Figure 1D, Supplementary Figure 1D), mostly from patients with tumors or hydrocephalus (Supplementary Figure 1B). Data were collected from 108 patients aged 0 to 85 years (Figure 1B–C) from 485 human cortical layer 2/3 (L2/3) pyramidal cells. To confirm that the studied pyramidal cells originate from the L2/3, we measured the distance between the cell body and the L1 border (Berg et al., 2021). 35% of the cells recovered their soma, with a distance of 130.66 ± 134.63 μm from the L1 border (Fig 1E). The data set was divided into seven age groups (Bethlehem et al., 2022; Kang et al., 2011): infant: < 1 year, early childhood: 1–6 years, late childhood: 7–12 years, adolescence: 13–19 years, young adulthood: 20–39 years, middle adulthood: 40–59 years, late adulthood: ≥ 60 years (Figure 1A). We evaluated the voltage deflections induced by negative and positive current injections and extracted subthreshold membrane features such as resting membrane potential, input resistance, time constant (tau), and sag ratio in 475 cortical pyramidal cells from 99 patients. We found that the subthreshold features from samples of infant significantly different from those from other age groups (Figure 2A–D; resting membrane potential: P =3.53×10^−8^, input resistance: P = 1.29×10^−16^, tau: P = 1.31×10^−15^, sag ratio: P = 5.2×10^−4^, Kruskal-Wallis test). The resting membrane potential was significantly more positive in the first year of life than in the other age groups. Before adulthood a slight decrease was observed in the resting membrane potential across the groups (infant: n = 72, −60.64 ± 9.86 mV; early childhood: n = 28, −65.44 ± 6.82 mV; late childhood: n = 45, −65.17 ± 6.68 mV; adolescence: n = 54, −67.86 ± 5.94 mV; young adulthood: n = 89, −68.69 ± 5.54 mV; middle adulthood: n = 56, −65.14 ± 5.5 mV; late adulthood: n = 113, −67.05 ± 7.86 mV) (Figure 2A). Input resistance, tau and sag ratio were measured on voltage deflections elicited by injecting negative (−100 pA) current steps into the cells. A significant decrease in input resistance (infant: n = 72, 257.25 ± 188.06 MΩ; early childhood: n = 28, 75.27 ± 37.5 MΩ; late childhood: n = 45, 74.61 ± 34.29 MΩ; adolescence: n = 54, 64.79 ± 41.82 MΩ; young adulthood: n = 89, 70.85 ± 46.9 MΩ; middle adulthood: n = 56, 90.14 ± 54.37 MΩ; late adulthood: n = 113, 81.14 ± 46.36 MΩ) was observed with the largest reduction after the first year of age (P = 1.88×10^−6^, Kruskal–Wallis test with post-hoc Dunn test) (Figure. 2B). The membrane time constant also decreased significantly in the older groups compared to the infant group, after infancy we found more conserved mean values of membrane time constant into older age (infant: n = 72, 23.88 ± 14.7 ms; early childhood: n = 28, 8.49 ± 3.08 ms; late childhood: n = 45, 9.73 ± 4.24 ms; adolescence: n = 54, 8.99 ± 3.71 ms; young adulthood: n = 89, 8.76 ± 6.33 ms; middle adulthood: n = 56, 8.53 ± 4.24 ms; late adulthood: n = 113, 10.39 ± 6.63 ms) (Figure 2C). The ratio of the maximal deflection and the steady-state membrane potential during a negative current step (sag ratio) is significantly higher in late adulthood than in the early stages of life (infant: n = 72, 0.079 ± 0.87; early childhood: n = 28, 0.075 ± 0.058; late childhood: n = 45, 0.082 ± 0.092; adolescence: n = 54, 0.086 ± 0.076; young adulthood: n = 89, 0.09 ± 0.084; middle adulthood: n = 56, 0.092 ± 0.073; late adulthood: n = 113, 0.12 ± 0.1) (Figure 2D). Note that the high variance of the infant group data (e.g. resting membrane potential, input resistance, Tau) are due to the dynamic change over the 0–1 year period (Supplementary Figure 3).

### Suprathreshold properties across age-groups

**Figure 3.**
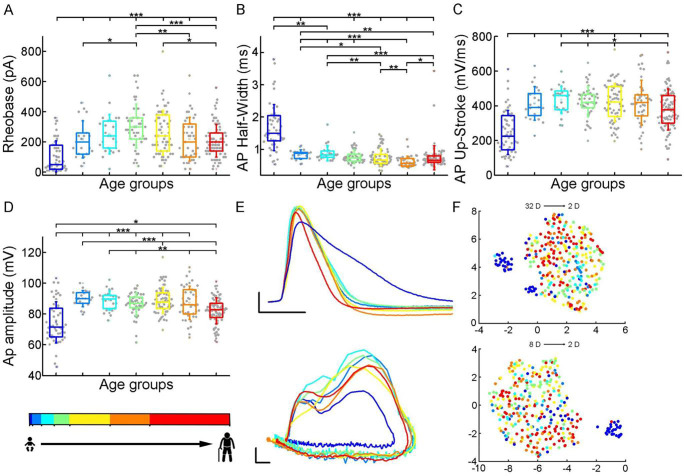
Age related differences in the action potential kinetics. (A-C) Boxplots show differences in rheobase (A), action potential half-width (B), action potential up-stroke (C), and action potential amplitude (D) between the age groups. Asterisks indicate statistical significance (* P < 0.05, ** P < 0.01, *** P < 0.001). (E) Representative action potentials aligned to threshold potential onset (scale: x axis: 1ms, y axis: 20 mV) (top) and phase plots of the representative APs (scale: x axis: 10 mV, y axis: 100 mV/ms) (bottom). (F) Uniform Manifold Approximation and Projection (UMAP) of 32 (top) and 8 selected electrophysiological properties (resting Vm, input resistance, tau, sag ratio, rheobase, AP half-width, AP up-stroke and AP amplitude) (bottom) with data points for 331 cortical L2/3 pyramidal cells, colored with the corresponding age groups.

To initiate action potentials we injected positive current steps increased by 20 pA into the cells and recorded various types of input-output transformations. We extracted 25 features from different action potentials (AP) and firing patterns and assessed active membrane properties from recordings filtered for appropriate electrophysiological quality (see Methods). We found that the infant group differs most from the other age groups in several of the suprathreshold properties (Figure 3), but other trends are also apparent (rheobase current: P = 8.71×10^−12^, AP half-width: P = 9.57×10^−25^, AP up-stroke velocity: P = 1.63×10^−12^, AP amplitude: P = 2.24×10^−11^, Kruskal-Wallis test). For example, the average rheobase current, the minimum current that can trigger an action potential, is significantly lower in the early ages of life than in data collected from adolescence stage (infant: n = 51, 104.51 ± 103.18 pA, early childhood: n = 21, 218.1 ± 123.27 pA, late childhood: n = 25, 252.8 ± 131.17 pA, adolescence: n = 45, 306.22 ± 147.21 pA, young adulthood: n = 63, 262.9 ± 148.06 pA, middle adulthood: n = 43, 219.07 ± 145.52 pA, late adulthood: n = 83, 207.71 ± 107.83 pA; infant vs. adolescence P = 4.15×10^−13^,Kruskal–Wallis test with post-hoc Dunn test). Further on the age scale we found that rheobase current was increased with age reaching a maximum value at the adolescent ages then declining to a significantly lower level (adolescent vs. late adulthood, P = 5.23×10^−4^, Kruskal–Wallis test with post-hoc Dunn test, Figure. 3A). The action potential (AP) half-width averages among a declining trend through the groups of age (Figure 3B) varied considerably across age groups forming significant differences between childhood and adulthood ages (infant: n = 51, 1.68 ± 0.71 ms; early childhood: n = 21, 0.84 ± 0.15 ms; late childhood: n = 25, 0.88 ± 0.24 ms; adolescence: n = 45, 0.78 ± 0.22 ms; young adulthood: n = 63, 0.76 ± 0.27 ms; middle adulthood: n = 43, 0.62 ± 0.17 ms; late adulthood: n = 83, 0.74 ± 0.38ms). Action potential up-stroke velocities were significantly slower in the infant APs than in all the other ages (infant: n = 51, 247.43 ± 127.27 mV/ms; early childhood: n = 21, 413.68 ± 97.11 mV/ms; late childhood: n = 25, 434.75 ± 78.32 mV/ms; adolescence: n = 45, 424.69 ± 87.58; young adulthood: n = 63, 418.49 ± 110.33 mV/ms; middle adulthood: n = 43, 419.15 ± 129.29 mV/ms; late adulthood: n = 83, 379.22 ± 118.45mV/ms) (Figure 3C). The amplitude of the elicited APs also showed age-dependent differences between age groups; in the first year of life, the amplitude of APs were significantly lower than in other age groups. In adulthood, a significant decrease with age was observed (infant: n = 51, 74.62 ± 13.4 mV; early childhood: n = 21, 90 ± 5.23 mV; late childhood: n = 25, 88.82 ± 6.35 mV; adolescence: n = 45, 86.31 ± 7.36 mV; young adulthood: n = 63, 88.81 ± 8.52 mV; middle adulthood: n = 43, 87.28 ± 10.77 mV; late adulthood: n = 83, 82.02 ± 8.49 mV) (Figure 3D). Electrophysiological differences markedly separated the infant group from the older age groups shown on UMAP projection (Figure 3F, Supplementary Figure 2).

**Figure 4.**
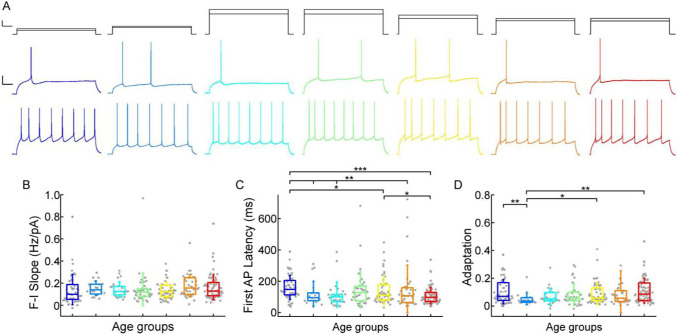
Age-dependency of the AP firing pattern parameters (A) Representative membrane potential responses to an 800 ms long rheobase (middle) (left to right: infant, early childhood, late childhood, adolescence, young adulthood, middle adulthood, late adulthood), and increased current steps (bottom) Colored respectively to the age groups. Scale bar top: 1ms, 100 pA, bottom: 1ms, 20mV. (B-D) Boxplots show changes across the age groups in f-I slope (B), first AP latency (C), and adaptation of APs (D). Asterisks indicate statistical significance (* P < 0.05, ** P < 0.01, *** P < 0.001).

Next, we investigated how somatic current inputs are transformed to action potential output by evaluating the firing patterns of cells evoked by injecting prolonged positive current steps (Figure 4A). The neurons (n = 300) were regular spiking cells with moderate adaptation, we excluded cells showing initial duplet or triplet action potential bursts (10.7% of all neurons). The slope of the firing frequency versus the current curve (f-I slope) showed no significant difference across the age groups (P = 0.1, Kruskal-Wallis test; infant: n = 51, 0.14 ± 0.14 Hz/pA; early childhood: n = 20, 0.15 ± 0.066 Hz/pA; late childhood: n = 25, 0.14 ± 0.07Hz/pA; adolescence: n = 39, 0.15 ± 0.15 Hz/pA; young adulthood: n = 54, 0.13 ± 0.08 Hz/pA; middle adulthood: n = 37, 0.18 ± 0.1 Hz/pA; late adulthood: n = 74, 0.16 ± 0.11 Hz/pA) (Figure 4B). Age related difference was observed in the latency of the first AP during rheobase current injection (First AP latency) (infant: n = 51, 161.26 ± 76.81 ms; early childhood: n = 20, 122.16 ± 78.79 ms; late childhood: n = 25, 115.29 ± 81.27 ms; adolescence: n = 39, 145.93 ± 115.13; young adulthood: n = 54, 140.89 ± 87.88; middle adulthood: n = 37, 151.83 ± 151.19; late adulthood: n = 74, 108.88 ± 52.98 ms), in the first year of life the latency of the first spike is significantly higher than all the other groups of age (P = 1.47×10^−3^, Kruskal-Wallis test), which is the most prominent with the oldest age group (P = 3.47×10^−5^, Kruskal–Wallis test with post-hoc Dunn test) (Figure 4C). The adaptation of the AP frequency response to the same current injection stimulus also showed differences (P = 0.043, Kruskal-Wallis test) between the younger and the groups older than 13 years patients, we found the lowest adaptation values in early childhood (infant: n = 51, 0.1 ± 0.087; early childhood: n = 20, 0.051 ± 0.042; late childhood: n = 25, 0.068 ± 0.06; adolescence: n = 38, 0.081 ± 0.076; young adulthood: n = 54, 0.09 ± 0.077; middle adulthood: n = 37, 0.102 ± 0.152; late adulthood: n = 74, 0.11 ± 0.088) (Figure 4D).

Previous research has shown that the biophysical properties of the human pyramidal cells show depth-related correlations throughout the L2/3 (Berg et al., 2021). Although there are some deeper cells in our dataset, the majority comes from the upper region of the L2/3. We compared the electrophysiological characteristics according to their depth from the border of L1 and L2 to exclude the possibility that the biophysical differences we found were a result of depth dependence. We did not find any overall differences related to distance of the soma from the L1 border within the age groups with a few exceptions. For example, the values of input resistance (P = 0.02, Mann-Whitney test) and AP up-stroke velocity (P = 0.04, Mann-Whitney test) differ significantly in the middle adulthood group. We found a significant difference in AP amplitude (P = 0.02, Mann-Whitney test) and adaptation (P = 0.009, Mann-Whitney test) in the adolescence age group (Supplementary Figure 9–11).

### Morphological features of layer 2/3 pyramidal cells in different stages of life.

**Figure 5.**
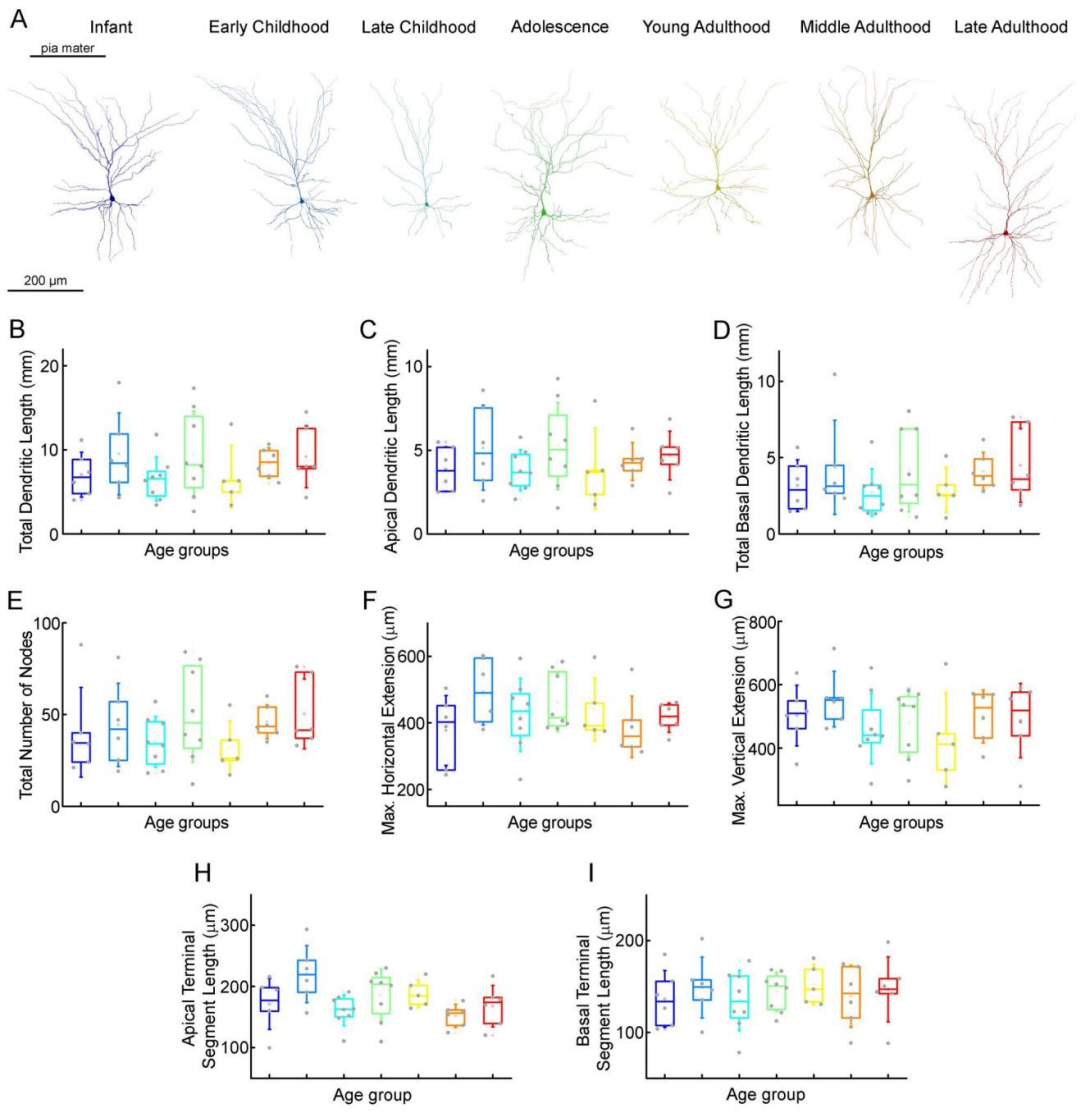
Morphological features of layer 2/3 pyramidal cells in different stages of life (A) Representative reconstructions of L2/3 pyramidal cells (from left to right) from infant (n = 6), early childhood (n = 6), late childhood (n = 8), adolescence (n = 8), young adulthood (n = 5), middle adulthood (n = 6), and late adulthood (n = 6) patients. (B-I) Boxplots show summarized data from all the reconstructed cells (Suppl. fig. 8) of total dendritic length (B), apical dendritic length (C), total basal dendritic length (D), the total number of nodes on the apical and basal dendrites (E), the maximal horizontal (F), and the maximal vertical (G) extension of dendrites, the average length of the apical (H) and basal (I) terminal dendritic segments.

To investigate possible morphological differences between the age groups, we filled the pyramidal cells with biocytin during recordings. Only neurons with no signs of deterioration and with complete apical dendrites and no signs of truncated dendritic branches or tufts were considered for morphological analysis. 45 pyramidal cells (Figure Supplement 8) were reconstructed in 3D at ages 0–73 years (infant n = 6, early childhood n = 6, late childhood n = 8, adolescence n = 8, young adulthood n = 5, middle adulthood n = 6, late adulthood n = 6). Figure 5A shows examples of the reconstructed pyramidal cells. We did not detect significant change in total dendritic length (P = 0.5, Kruskal-Wallis test; infant: 7.05 ± 2.66 mm; early childhood: 9.52 ± 4.87 mm; late childhood: 6.52 ± 2.62 mm; adolescence: 9.42 ± 5.19 mm; young adulthood: 6.79 ± 3.7 mm; middle adulthood: 8.43 ± 1.82 mm; late adulthood: 9.18 ± 3.69 mm) (Figure 5B), apical dendritic length (P = 0.7, Kruskal-Wallis test; infant: 3.88 ± 1.32 mm; early childhood: 5.16 ± 2.55 mm; late childhood: 3.81 ± 1.23 mm; adolescence: 5.25 ± 2.6 mm; young adulthood: 3.91 ± 2.42 mm; middle adulthood: 4.32 ± 1.13 mm; late adulthood: 4.69 ± 1.46 mm) (Figure 5C), or basal dendritic length (P = 0.46, Kruskal-Wallis test; infant: 3.17 ± 1.69 mm; early childhood: 4.36 ± 3.08 mm; late childhood: 2.72 ± 1.55 mm; adolescence: 4.17 ± 2.72 mm; young adulthood: 2.89 ± 1.47 mm; middle adulthood: 4.11 ± 1.24 mm: late adulthood: 4.49 ± 2.42 mm) (Figure 5D) at different ages. To investigate dendritic complexity, we measured the total number of dendritic branching and found no significant developmental changes (P = 0.43, Kruskal-Wallis test; infant: 40.33 ± 24.42; early childhood: 44.33 ± 22.72; late childhood: 35.13 ± 13.84; adolescence: 50.38 ± 26.4; young adulthood: 31.8 ± 14.62; middle adulthood: 46 ± 9.27; late adulthood: 50.33 ± 19.01) (Figure 5E). We also found no significant differences in the size of dendritic branching when we measured the maximum horizontal (P = 0.48, Kruskal-Wallis test; infant: 376.98 ± 105.02 μm; early childhood: 492.91 ± 99.16 μm; late childhood: 423.59 ± 110.04 μm; adolescence: 460.76 ± 86.69 μm; young adulthood: 440.13 ± 93.87 μm; middle adulthood: 388.02 ± 92.16 μm; late adulthood 415.49 ± 43.74 μm) (Figure 5F) and vertical (P = 0.65, Kruskal-Wallis test; infant: 502.21 ± 95.51 μm; early childhood: 554.67 ± 87.17 μm; late childhood: 461.66 ± 111.28; adolescence: 479.9 ± 108.07 μm; young adulthood: 426.28 ± 148.8 μm; middle adulthood: 499.6 ± 83.2 μm; late adulthood: 486.89 ± 117.23 μm) (Figure 5G) extent of the reconstructed cells. We did not observe significant differences in the average length of the uncut terminal segments of apical (P = 0.052, Kruskal-Wallis test; infant: 171.2 ± 41.54 μm; early childhood: 220.2 ± 46.73 μm; late childhood: 160.79 ± 25.08; adolescence: 185.94 ± 42.14 μm; young adulthood: 188.27 ± 22.74 μm; middle adulthood: 151.8 ± 18.75 μm; late adulthood: 167.71 ± 34.06 μm) (Figure 5H) and basal (P = 0.96, Kruskal-Wallis test; infant: 136.49 ± 30.9 μm; early childhood: 148.96 ± 33.11 μm; late childhood: 134.74 ± 32.88 μm; adolescence: 144.32 ± 20.9 μm; young adulthood: 152.1 ± 22.04 μm; middle adulthood: 139.25 ± 33.66 μm; late adulthood: 146.94 ± 35.46 μm) (Figure 5I) dendrites of the cells across the age groups.

**Figure 6.**
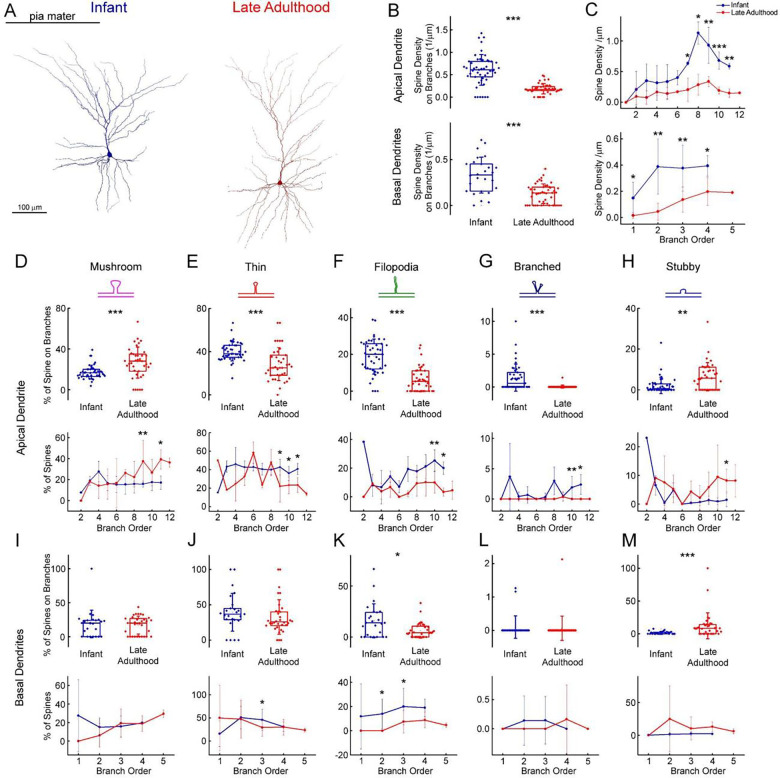
Comparison of the number of dendritic spines between an infant and a late adulthood aged sample (A) Anatomical 3D reconstruction of human L2/3 pyramidal cells from the infant (left), and the late adulthood (right) age groups. (B) Boxplots of the spine densities on the apical (top), and basal (bottom) dendritic branches from the infant (blue) and late adulthood (red) L2/3 pyramidal cells shown on panel (A). (C) Graphs showing the distribution of dendritic spine density as a function of the branch order, the infant pyramidal cell shown with blue, the late adulthood pyramidal cell with red, on the apical (top) and on the basal (bottom) dendrites. (D-H) The plots show the distribution of mushroom (D), thin (E), filopodium (F), branched (G), and stubby (H) dendritic spine types on the apical dendrites of the reconstructed infant (blue) and late adult (cell) pyramidal cells. Top, schematic representation of the examined dendritic spine types. Center, age dependent distribution of spine types. Bottom, spine distributions along branch orders. Asterisks indicate significance (* P < 0.05, ** P < 0.01, *** P < 0.001) (I-M) Same as D-H but on basal dendrites.

We identified and labeled each spine on n = 2 fully 3D-reconstructed cells to analyze the distribution of dendritic spines (Figure 6). We compared the spine density of two selected pyramidal cells derived from an infant (83 days old) and a patient in late adulthood (65 years old). We found that the total spine density was higher (P = 4.6×10^−14^, Mann-Whitney test) and also the spine density of both apical (P = 8.02×10^−10^, Mann-Whitney test) and basal (P = 1.12×10^−5^, Mann-Whitney test) dendrites was higher in the infant than in the late adult (Figure 6B). To evaluate the age-dependence of spine morphology, we classified the spines into different phenotypes based on their morphological characteristics, specifically distinguishing between mushroom-shaped, thin, filopodial, branched, and stubby spines (Figure 6D–H). Spines with large spine heads were classified as mushroom-shaped, those with small heads as thin, and long protrusions were distinguished as filopodial. Those that did not have peduncles were classified as stubby, spines with two heads emerging from the same spot were called branched spines (Figure 6D–H) (Luebke et al., 2015). Only fully visible spines were included in the classification analysis. The composition of spine types varies between the two age groups, mushrooms are present at a higher percentage on apical branches in late adulthood (P = 1.85×10^−4^, Mann-Whitney test), but not on basal processes (P = 0.83, Mann-Whitney test) (Figure 6D,I). In contrast, thin spines and filopodia are present in significantly higher numbers on the apical (Figure 6E–F) (thin spines: P = 4.55×10^−5^, two sample t-test; filopodia: P = 3.09×10^−8^, Mann-Whitney test) and basal (filopodia: P = 0.027, Mann-Whitney test) (Figure 6J–K) dendritic branches of the infant. Apical infant branches had a significantly higher percentage of branched spines (P = 1.46×10^−6^, Mann-Whitney test) (Figure 6G), which were present in modest numbers on both cells but not on the basal ones (P = 0.42, Mann-Whitney test) (Figure 6l). Stubby spines were also more prevalent on the elderly pyramidal cell, either on the apical (P = 4.34×10^−3^, Mann-Whitney test) or basal (P = 3.21×10^−4^, Mann-Whitney test) processes (Figure 6H,M). On the apical dendrites, the number of spines increased with the order of branching, regardless of morphology type, and generally reached a significant difference at the higher order apical branches (Figure 6D–H).

Cortical tissue was dissected during neurosurgical procedures for various pathologies, but not to the same extent in the different groups. Specimen collection in adult/elderly patients was mostly for tumor resection, whereas in children hydrocephalus was the most common reason for brain surgery (Supplementary Figure 1). To determine the difference in medical condition could contribute to age-related differences in cellular features, we compared the extracted electrophysiological and morphological features based on the medical condition in the different age groups. It should be noted that all circumstances of surgical steps, tissue dissection, transport (time, media, temperature etc.) and cutting procedure remained the same in the different conditions. When comparing passive electrophysiological properties, we found no significant differences between the different medical conditions in the age groups from infancy to middle adulthood, only time constants were found to be significantly lower in the hydrocephalus patients than in the tumor patients in the young adulthood (P = 0.048, Mann-Whitney test) and late adulthood groups (P = 0.01, Mann-Whitney test) (Supplementary Figure 4). Comparing the action potential kinetics and firing pattern-related parameters between the different pathology groups we found no overall differences with some sporadic exceptions. For example, the parameters of AP half-width in young adulthood (P = 0.01, Mann-Whitney test) and the values of rheobase in early childhood (P = 0.04, Mann-Whitney test) differed significantly between the different pathologies (Supplementary Figure 5). Regarding the firing pattern-related properties we found significant changes in the infant F-I slope (P = 0.02, Mann-Whitney test), first AP latency (P = 0.006, two sample t-test) and adaptation (P = 0.002, Mann-Whitney test) parameters. Also, in the late adulthood group the F-I slope (P = 0.04, Mann-Whitney test), the first AP latency (P = 0.02, Mann-Whitney test) and the adaptation (P = 0.047, Mann-Whitney test) were significantly different between the hydrocephalus and tumor patients (Supplementary Figure 6). Further comparison of morphological features revealed no statistical difference between tumor and hydrocephalus groups (Supplementary Figure 7).

## Discussion

In this study, using the whole-cell patch-clamp technique and 3D reconstructions, we have studied the differences of human cortical L2/3 pyramidal cells at various stages of life. We found that during the lifespan the most significant changes take place early in life during the first year in most of the biophysiological characteristics of human pyramidal cells (see Supplementary Figure 3). We recorded from n = 457 human cortical excitatory pyramidal cells from the supragranular layer from birth to 85 years. Most differences in sub- and suprathreshold features were found in the youngest age groups. There were particular differences in resting membrane potential, input resistance, time constant, rheobasic current, AP halfwidth and AP upstroke velocity. Other age groups that differed the most from other age groups were the oldest (60 – 85 years) with modest differences in sag ratios. In our morphological analysis, we found no significant changes in the overall apical and basal dendritic dimensions across different ages. When evaluating the number of spines in two anatomical reconstructions (from the infant and late adulthood groups) we found a higher overall spine density in the infant than in the elder sample consisting of mainly branched and thin spines and filopodia. In contrast, stubby and mushroom-shaped spines were more prevalent in the older pyramidal cell.

The nervous system experiences a multitude of plastic changes throughout an individual’s lifetime. These changes extend from the early stages of development and continue through the gradual degenerative processes that come with old age. Although precise intrinsic cellular modifications linked to aging remain somewhat elusive, there is an observable morphological transformation during early development. As the nervous system matures, dendritic branching (Kasper et al., 1994; McAllister, 2000) and cell body size increases (Zhang, 2004). Also, learning and experience increase the extent of dendritic branching (Greenough & Volkmar, 1973; Holloway, 1966) and dendritic spines (Globus et al., 1973). An extensive body of research has shown that synaptic plasticity induces formation of new dendritic spines and the enlargement of existing spines (Kastellakis et al., 2023). These modifications may affect the input-output relation of a neuron as model studies suggest that dendritic geometry influences electrical and firing properties (Eyal et al., 2014; Mainen & Sejnowski, 1996; van Elburg & van Ooyen, 2010; Vetter et al., 2001). Furthermore, in parallel to the changes in morphological complexity there is ample experimental evidence that the maturation of ion channels and the change in increasing channel density in the neuronal membrane also play a role in influencing the electrical properties of neurons (Connors, 1994; Huguenard et al., 1988). These findings point to a close causal relationship between structure and electrophysiological properties at cellular level.

### Lifespan changes in electrophysiological properties

The intrinsic electrophysiological properties of a neuron are determined by several factors: the resistivity of the cytoplasm and membrane, the membrane capacitance, and the shape of the soma and dendrites. The basic passive parameters we recorded, such as the resting membrane potential, the input resistance, and the membrane time constant show a dramatical change in the first year. This change is consistent with data found in previous studies on neocortical pyramidal cells from rodent models (Kasper et al., 1994; Kroon et al., 2019; McCormick & Prince, 1987; Picken Bahrey & Moody, 2003; Zhang, 2004; Zhu, 2000) and on xenotransplated human cortical neurons (Linaro et al., 2019). We found a progressive shift of the resting membrane potential from −60 mV to −68 mV in the first part of the lifespan (<40 years), which could be due to the age-dependent change in ion channel composition (Picken Bahrey & Moody, 2003). The input resistance and time constant showed a sudden and sharp decrease between the first and second age groups, indicating the most dramatic changes in the infant brain followed by a generalized and slower decrease. As pyramidal cells mature, the size and volume of the cell body increase (Petanjek et al., 2019; Zhu, 2000) which correlates with the input resistance, meaning that a small membrane area has a higher input resistance (Luhmann et al., 2000). In addition to the increase in cell surface area, the increase in the expression of certain potassium leak channels, e.g. from the two-pore domain potassium channel (KCNK) family, can also influence the input resistance during cell maturation (Aller & Wisden, 2008; Goldstein et al., 2001). Similar reasons can lead to a decrease in the membrane time constant. The membrane time constant is the product of the specific input resistance and the specific membrane capacitance. Assuming constant ~1 pF/cm^2^ membrane capacitance (Beaulieu-Laroche et al., 2018; Oláh et al., 2024), the decrease is likely also due to a change in specific membrane resistance. The result of developmental changes in membrane passive parameters during early development improves spike timing precision and temporal responsiveness in the cortical microcircuit (Doischer et al., 2008; Oswald & Reyes, 2011). However, in our human samples at later ages (>40 years) that we associate with the aging brain, the previous trends of changes in intrinsic characteristics are reversed. Input resistance and time constant increase, which may be associated with the decline in KCNK expression in the aging brain (Erraji-Benchekroun et al., 2005). These membrane parameters are also generally higher in studies in rhesus monkeys (Moore et al., 2023), except for the resting membrane potential, which is not different from that of young cortical L2/3 (Chang, 2005) or L5 pyramidal cells (Luebke & Chang, 2007) in the brain. However, in our samples, we found a positive shift in the resting potential between the middle- and old-age groups by a few millivolts. It should be noted that this discrepancy may be due to the fact that the age groups in our work are not the same as the works mentioned.

Changes in resting membrane potential and input resistance will add up to produce a rising and then falling curve of the averaged rheobasic current, with the value three times higher in the adolescent group than in the youngest group and 1.5 times higher than in late adulthood. Our results on increased rheobase during early life are consistent with previous studies in rodent cortical L3 and 5 pyramidal cells (Kroon et al., 2019; Perez-García et al., 2021; Popescu et al., 2021) and with findings regarding aging in rhesus monkey cortical neurons from rhesus monkeys (Moore et al., 2023).

When we assessed the voltage sag ratio with age we found a consistent increase that peaked in late adulthood. The voltage sag that occurs during membrane hyperpolarization is the result of activation of the hyperpolarization-activated cyclic nucleotide-gated (HCN) current, also known as the h-current. Similar to our findings the age-dependent increase in sag deflection has been observed in human cortical pyramidal cells (Guet-McCreight et al., 2023; Kalmbach et al., 2018) which is consistent with studies by Wang et al. (Wang et al., 2007, 2011) that have linked inhibited HCN activation to working memory deficits in the elderly.

The electrical output of neurons is driven by the composition of neuronal molecules and ion channels whose expressions and distributions change most dynamically in two periods, early development, and the aging brain. This dynamic essentially produced an inverted U-shaped curve in the distribution of the active electrical properties over the lifespan in our dataset, an increase in the first year, a relatively stable middle phase, and then a return in older age groups. The origin of these changes is mainly due to changes in the distribution of sodium and potassium channels. During development, there is evidence that the number of voltage-gated Na^+^ channels increases in rodents (Huguenard et al., 1988; Picken Bahrey & Moody, 2003) and in humans (Erraji-Benchekroun et al., 2005), which may accelerate the kinetics of the action potential (up-stroke) and, similar to our results, increase the amplitude of the action potential (Etherington & Williams, 2011; Kroon et al., 2019; McCormick & Prince, 1987; Perez-García et al., 2021; Zhang, 2004). During aging, however, regulation is disrupted by the change in channel expression, which can lead to altered conduction rate of ionic currents. In brain senescence, the amplitude of the action potential and a slowing of the action potential (Luebke & Chang, 2007) have been shown to be similar to our data, which may be caused by the decreased expression of voltage-gated Na^+^ channels (Erraji-Benchekroun et al., 2005).

We examined the firing patterns of pyramidal cells at different ages by comparing the F-I slope, and we found that the patterns are fairly conserved across ages. In rodent developmental studies, a decrease in F-I slope was found only in the first two weeks after birth (McCormick & Prince, 1987; Zhang, 2004), which time window is not represented in our data. The latency of the first spike is influenced by the time-constant and specific ion channels, like the transient (or A-type) K^+^ current and the transient Ca^2+^ current (or T-type) (Molineux et al., 2005). The same ion channels are involved in burst firing at the onset of stimulation. Note that in our sample only ~10 % of pyramidal cells showed burst firing at the beginning of stimulation, but it is known that burst firing is more frequent from layer 3 onwards at depth (Berg et al., 2021). The presence of A-current has been found in immature pyramidal neurons from sensorimotor areas in rodent studies (McCormick & Prince, 1987), and the density of T-type currents also remains unchanged during the development of the visual cortex (Horibe et al., 2014). It has been shown that the expression of the T-type calcium channel family (CACNG3) decreases during aging (Erraji-Benchekroun et al., 2005). However, it must be mentioned that the differences in the expression of all subtypes of the Kv4 (A-type) and Ca_V_3.x (T-type) channel families during brain development and aging have not been systematically described.

### Properties of dendritic trees and spine distribution with age

Transcriptomic analyses of the human neocortex show relatively stable expression patterns of genes for dendritic and synaptic development after about 1 year of age (Kang et al., 2011). In early postmortem studies human pyramidal neurons were studied for morphological changes as a function of age. In these studies, using Golgi staining methods, it was found that the dendritic trees of infant layer 2/3 pyramidal cells are well developed (Mrzljak et al., 1991). In the human prefrontal cortex after birth, the dendritic tree of layer 3 pyramidal neurons reaches its structurally mature form after about 3 months (Petanjek et al., 2008). Thereafter, it is assumed that a smaller increase in dendritic length reaches its final form between about 7.5–12-months (Koenderink & Uylings, 1995; Petanjek et al., 2008). In our study, we observed stable patterns of apical and basal dendrite lengths and dendritic tree complexity across the lifespan. Even between the early postnatal groups (infancy vs. early childhood), no significant difference in dendritic tree size was observed. This can be explained by the influence of various factors, such as the large difference between individual subjects or the subtype specific dendritic morphology of pyramidal cells (Berg et al., 2021) or different maturation curves of neurons from different brain regions (Elston & Fujita, 2014) or cortical layers (Petanjek et al., 2008).

Neurons exhibit a phase of synaptogenesis overproducing synapses lasting months or years in the human cortex (Huttenlocher, 1979; Huttenlocher et al., 1982; Mrzljak et al., 1991; Petanjek et al., 2011), followed by dendritic spine/synapse pruning that is reported to last more than a decade in the cortex (Benavides-Piccione et al., 2013; Coskren et al., 2015; Jacobs et al., 1997; Mavroudis et al., 2015; Petanjek et al., 2011). Our results from the comparison of pyramidal cells from an infant (83 days old) and a late adult (65 years old) also show that the overall number of dendritic spines decreases with increasing age. When evaluating the distribution of spine shapes in young and old pyramidal cells, we found spines with larger heads (mushroom shape) in a greater number in the dendrite of old pyramidal cells, which are mature synapses. The size of the head is an indicator of the size of the postsynaptic density, the number of glutamate receptors, and the strength of the synapse (Bourne & Harris, 2008; Matsuzaki et al., 2001; Nusser et al., 1998). In the developing nervous system, however, the filopodia, thin dendritic protrusions without a head, are the most characteristic type of spines. The density of filopodial spines was higher on the dendrites of infant pyramidal cells. In the older cells, we still found filopodia, which are the silent precursor of active synapses (Vardalaki et al., 2022). The change in the ratio of subtypes from young to old age is well established and is associated with the basis for changes in cognitive function during aging (Dumitriu et al., 2010; Jacobs et al., 1997; Luebke et al., 2015).

The complexity of human brain activity and cognitive abilities increases with development and decreases with aging, resulting in an inverted U-shaped curve across the lifespan (Craik & Bialystok, 2006). Cognitive functions depend on many age-dependent factors, such as the density and specificity of synaptic connections formed by synaptic pruning and plasticity, or the degree of myelination and white matter maturation and there is also an age-related modulation of neurotransmitters, hormones, ion transporters and receptors (Luna et al., 2015). Here we have shown that pyramidal cells become less excitable and temporarily more precise during development by changing their intrinsic functional properties and with aging these changes occur somewhat in opposite directions making the intrinsic parameters change symmetrically. In addition, some of the changes are asymmetrical either occur with development or with aging, such as the resting membrane potential which mostly changes in young ages or the ratio of sag, which is shifted most in old age. These changes in the intrinsic properties of cells during the first and last stages of life also contribute to the input-output functions of a neuron and ultimately to the age-related development of cognitive abilities.

## Materials and methods

### Slice preparation

Experiments were performed according to the Declaration of Helsinki with the approval of the University of Szeged Ethical Committee. Prior to surgery, the patients provided written consent for all tissue material. We used human cortical tissue that had to be surgically removed from patients (n = 63 female, n = 45 male) as part of the treatment for tumors, hydrocephalus, apoplexy, cysts, and arteriovenous malformation. Anesthesia was induced with intravenous midazolam and fentanyl (0.03 mg/kg, 1–2 μg/kg, respectively). A bolus dose of propofol (1–2 mg/kg) was administered intravenously. The patients received 0.5 mg/kg rocuronium to facilitate endotracheal intubation. The trachea was intubated, and the patient was ventilated with a mixture of O_2_-N_2_O at a ratio of 1:2. Anesthesia was maintained with sevoflurane at a care volume of 1.2–1.5. During the surgical procedure tissue blocks were removed from parietal (n = 22), temporal (n = 34), frontal (n = 42), and occipital (n = 10) regions, the resected tissue blocks were immediately immersed in ice-cold solution. Slices were cut perpendicular to the pia mater at a thickness of 320 μm with a vibrating blade microtome (Microm HM 650 V) in ice-cold solution (in mM) 75 sucrose, 84 NaCl, 2.5 KCl, 1 NaH_2_PO_4_, 25 NaHCO_3_, 0.5 CaCl_2_, 4 MgSO_4_, 25 D(+)-glucose, saturated with 95% O2 and 5% CO2. The slices were incubated in the same solution for 30 minutes at 36°C following that the solution was changed to (in mM) 130 NaCl, 3.5 KCl, 1 NaH_2_PO_4_, 24 NaHCO_3_, 1 CaCl_2_, 3 MgSO_4_, 10 D(+)-glucose, saturated with 95% O_2_ and 5% CO_2_, the slices were kept in it until use.

### In vitro electrophysiological recordings

Somatic whole-cell current-clamp recordings were obtained at ~36 °C in solution containing (in mM) 130 NaCl, 3.5 KCl, 1 NaH_2_PO_4_, 24 NaHCO_3_, 3 CaCl_2_, 1.5 MgSO_4_, 10 D(+)-glucose, from layer 2/3 pyramidal cells visualized by infrared differential interference contrast (DIC) video microscopy equipped with micromanipulators (Luigs and Neumann, 652 Ratingen, Germany) and HEKA EPC 9&10 patch clamp amplifier (HEKA Elektronik GmbH, Lambrecht, Germany). Micropipettes (3–5 MΩ) were filled with intracellular solution containing (in mM) 126 potassium-gluconate, 4 KCl, 4 ATP-Mg, 0.3 GTP-Na_2_, 10 HEPES, 10 phosphocreatine, and 8 biocytin (pH 7.20; 300 mOsm). After whole-cell configuration was obtained, stepwise currents were injected to measure the evoked sub- and suprathreshold membrane potential properties. To calculate passive membrane properties (resting membrane potential, input resistance, time constant and sag) either cells with series resistance (Rs): 22.85 ± 9.04 MΩ (ranging between −4.55 MΩ and 56.76 MΩ) and 0 pA holding current (n = 154), or cells with holding current > −50 pA (−7.46 ± 28.56 pA, min: −49.89 pA, max: 59.68pA) and Rs < 30 MΩ (18.96 ± 6.48 MΩ) (n = 23) were used. For the analysis of high frequency action potential features (AP half-width, AP up-stroke velocity, AP amplitude and rheobase) cells with Rs < 30 MΩ (n = 331 cells with Rs 19.2 ± 6.6 MΩ) and holding current > −50pA (n = 308 with 0 pA holding current and Rs: 19.22 ± 6.59 MΩ, n = 23 with holding current: −7.46 ± 28.56 pA and Rs: 18.96 ± 6.48 MΩ) were used. To measure firing pattern properties (first spike latency, F-I slope and adaptation) cells with Rs > 30 MΩ, holding current < −50 pA and initial burst firing (inter spike interval < 20 ms) were excluded. N = 300 cells were used (n = 277 with 0 pA holding current with Rs: 19.32 MΩ ± 6.64, and n = 23 with holding current: −7.46 pA ± 28.56 pA (min: −49.89 pA, max: 57.68 pA) and Rs: 18.96 ± 6.48 MΩ, ranging between 1.79 MΩ and 28.87 MΩ).

### Data analysis

Electrophysiological features were measured from voltage responses elicited by 800 ms long current steps increasing by 20 pA from −100 pA. We analyzed the electrophysiological data with Fitmaster software (HEKA Elektronik GmbH, Lambrecht), and custom MATLAB (The Math Works, Inc.) scripts. The resting membrane potential of the neuron was measured directly after attaining the whole-cell configuration with no current injection (if a holding current was used during the recording we compensated the resting membrane potential with the injected current). To calculate input resistance, and time constant (tau) the mean of all hyperpolarizing current produced voltage steps was used. Subthreshold features included the input resistance and membrane time constant (tau) from response across or before hyperpolarizing steps, and sag ratio from the voltage response to −100 pA current injection. The rheobase current was defined as the minimal current that elicited the first spike. The up-stroke value was calculated as the mean of all the maximum values of dV/dt between the action potential onset and the action potential peak from each elicited APs of the cell. The F-I slope is the slope of the line fitted to the data of the AP firing frequency versus stimulus intensity. The first AP latency is defined as the duration until the first AP under the rheobasic current step. The adaptation of the elicited APs was calculated as the average adaptation of the interspike interval between consecutive APs. In addition to the electrophysiological parameters described above, 21 further parameters were calculated on the investigated cells, which are shown in Figure 3F UMAP (top panel): ratio of rebound, ratio of rebound and sag amplitude, average number of elicited APs per sweep, AP threshold, AP rise time (from AP threshold to AP peak), AHP amplitude, AHP length (duration of 0 (AHP minimum) to 90% of the AHP), average voltage value at the maximum of the AP dv/dt over all APs, average velocity value at the minimum of the AP dv/dt from all APs, average voltage value at the minimum of the AP dv/dt from all APs, average of AP maximum voltages, average interspike interval, AP adaptation, AP half-width adaptation, AP threshold adaptation, average of the interspike intervals in individual sweeps with at least two spikes, AP amplitude adaptation, AP half-width adaptation, AP threshold adaptation, AHP area (integral of the AHP from the minimum value to 90%, using trapezoidal method), ADP amplitude.

Analysis of morphological features was made by NeuroExplorer software (MBF Bioscience, Williston, VT, USA) and Origin 9 (OriginLab, Northampton, MA).

### Statistics

Data presented as the mean ± s.d. Normality was tested with the Lilliefors test, for statistical analysis, ANOVA with posthoc Bonferroni test, Kurskal-Wallis with posthoc Dunn test, for pairwise comparison two-sample t-test or Mann-Whitney test was used. Differences were accepted as significant if p < 0.05. The data are shown on boxplots, boxes indicate 25th, 50th (median), and 75th percentiles, rectangle represents the mean value, and whiskers indicate s.d.

### Histology and reconstruction

Slices were fixed in a fixative of 4% paraformaldehyde, 15% picric acid, and 1.25% glutaraldehyde in 0.1 M phosphate (PB, pH = 7.4) for at least 12 hours after electrophysiological recording. After multiple washes in 0.1 M PB, slices were cryoprotected in 10% then 20% sucrose solution in 0.1 M PB. The slices were frozen in liquid nitrogen and then thawed in PB. Slices were embedded in 10% gelatin and further sectioned into 70 μm thick sections. Sections were incubated in a solution containing conjugated avidin-biotin horseradish peroxidase (ABC; 1:100; Vector Labs) in Tris-buffered saline (TBS, pH = 7.4) overnight at 4°C. The enzyme reaction became visible by using 0.05% 3’3-diaminobenzidine tetrahydrochloride as a chromogen and 0.01% H_2_O_2_ as an oxidant. Sections were post-fixed with 1% OsO_4_ in 0.1 M PB. Following several washes with distilled water, sections were stained in 1% uranyl acetate, and dehydrated in an ascending series of ethanol. The sections were infiltrated with epoxy resin (Durcupan, Sigma-Aldrich) overnight and embedded on glass slides. After the electrophysiologically recorded cells have been visualized by DAB staining, 3D light microscopic reconstructions were carried out using the Neurolucida system (MBF Bioscience, Williston, VT, USA) with a 100× objective. The length of terminal segments was measured between the terminal tip of the dendrites and the last branching point before the terminal tip. Dendritic spine density on the dendritic branches was calculated as spine/μm between two bifurcations. The percentage of spines on branches was calculated as the percentage of the given spine type from all the types of spines on that branch.
