# Supplementary material for: Electrophysiology and Morphology of Human Cortical Supragranular Pyramidal Cells in a Wide Age Range
Source: bioRxiv. 2025 Jan 6:2024.06.13.598792. Originally published 2024 Jun 13. Preprint. [Version 2] doi: 10.1101/2024.06.13.598792 (PMC11195274; doi:10.1101/2024.06.13.598792)
Supplement: 1 [file NIHPP2024.06.13.598792V2-supplement-1.pdf]

bioRxiv preprint doi: https://doi.org/10.1101/2024.06.13.598792; this version posted January 6, 2025. The copyright holder for this preprint (which was not certified by peer review) is the author/funder, who has granted bioRxiv a license to display the preprint in perpetuity. It is made available under aCC-BY 4.0 International license.

Supplementary figures and tables

Age (years)	Sex	Brain region	Hemisphere	Surgical procedure
0.66	female	T	right	tumor removal
0.08	male	T	right	shunt
0.28	male	O	left	shunt
0.1	female	F	left	tumor removal
0.23	male	P	right	shunt
0.67	male	F	left	shunt
2	male	F	left	shunt
1	female	F	right	shunt
4	male	T	left	tumor removal
5	female	P	right	tumor removal
2	male	P	right	shunt
2	female	P	right	shunt
1	female	TO	left	shunt
7	male	F	right	tumor removal
7	male	P	right	tumor removal
10	male	F	left	tumor removal
10	female	F	left	tumor removal
11	female	F	right	tumor removal
11	male	T	left	shunt
10	male	T	right	other
13	female	F	right	tumor removal
16	male	PO	right	tumor removal
18	male	F	right	tumor removal
18	female	T	left	tumor removal
19	male	O	right	shunt
19	female	F	right	tumor removal
19	male	P	right	shunt
16	male	T	right	tumor removal
13	female	T	right	other
15	female	F	right	shunt
15	female	O	left	shunt
19	female	F	right	tumor removal
28	female	F	right	tumor removal
27	female	F	right	other
20	male	T	right	tumor removal
30	female	F	right	shunt
25	female	O	right	shunt
29	female	T	left	other
25	female	FT	left	tumor removal
23	female	T	right	other
26	female	F	right	shunt
30	male	F	left	tumor removal
21	female	F	right	shunt
35	male	T	right	tumor removal
35	female	TO	left	tumor removal
39	female	F	left	tumor removal
36	male	T	left	tumor removal
32	male	T	right	tumor removal
33	male	F	right	shunt
34	male	P	left	tumor removal
20	male	F	right	shunt
30	female	F	right	tumor removal

Age (years)	Sex	Brain region	Hemisphere	Surgical procedure
45	female	F	right	tumor removal
41	male	T	right	shunt
53	male	T	right	tumor removal
55	female	T	left	tumor removal
43	male	T	right	tumor removal
51	female	F	right	other
46	female	P	right	shunt
58	male	T	left	tumor removal
57	female	T	right	tumor removal
58	female	T	left	tumor removal
47	female	F	right	tumor removal
51	male	O	left	shunt
43	male	F	right	shunt
59	female	P	right	shunt
57	female	O	right	shunt
43	female	O	right	shunt
56	female	F	left	shunt
51	male	FT	left	tumor removal
49	female	F	right	tumor removal
40	female	P	right	tumor removal
49	male	O	right	tumor removal
70	male	T	left	tumor removal
72	female	T	right	tumor removal
81	female	O	right	shunt
63	male	F	right	tumor removal
65	male	T	right	tumor removal
69	female	P	left	other
70	female	P	right	shunt
77	male	P	right	shunt
79	female	P	right	shunt
63	female	T	right	tumor removal
64	male	T	left	tumor removal
69	female	O	right	shunt
66	female	F	right	tumor removal
75	female	O	left	shunt
78	female	T	right	shunt
60	female	T	right	tumor removal
60	female	F	right	tumor removal
62	female	F	right	shunt
62	female	T	right	tumor removal
62	female	P	left	tumor removal
63	female	F	right	tumor removal
63	female	O	right	tumor removal
63	female	F	left	tumor removal
63	female	F	right	shunt
64	male	F	right	tumor removal
76	male	F	right	tumor removal
85	male	FT	right	shunt
85	female	O	left	shunt
82	female	T	right	other
73	male	T	right	tumor removal
68	female	T	right	tumor removal

bioRxiv preprint doi: https://doi.org/10.1101/2024.06.13.598792; this version posted January 6, 2025. The copyright holder for this preprint (which was not certified by peer review) is the author/funder, who has granted bioRxiv a license to display the preprint in perpetuity. It is made available under aCC-BY 4.0 International license.

26	female	P	left	tumor removal
20	male	T	right	tumor removal
45	male	P	right	shunt
66	female	P	right	shunt
64	male	FP	left	tumor removal

Supplementary table 1. Patient metadata
Table showing the patients age, gender, brain region (F - frontal, T - temporal, P - parietal, O - occipital, PO - parieto-occipital, FT - fronto-temporal, TO - temporo-occipital, FP - fronto-parietal), hemisphere and the surgical procedure.

	Infant					Late adulthood				
	**#1 cell**	**#2 cell**	**#3 cell**	**Mean**	**SD**	**#1 cell**	**#2 cell**	**#3 cell**	**Mean**	**SD**
**Total spine number**	3039	5883	4443	4455	1422.04	1909	2977	1553	2146.33	741.07
**Apical spine number**	2352	3256	3568	3058.67	631.56	1237	1282	849	1122.67	238.07
**Basal spine number**	687	2627	875	1396.33	1069.93	672	1695	704	1023.67	581.61
**Mushroom**	477	970	762	736.33	247.5	629	1085	407	707	345.66
**Thin**	1055	2157	1666	1626	552.09	455	680	403	512.67	147.23
**Filopodia**	732	1431	936	1033	359.45	223	154	105	160.67	59.28
**Branched**	31	62	53	48.67	15.95	5	1	2	2.67	2.08
**Stubby**	67	99	84	83.33	16.01	83	247	134	154.67	83.93
**Not classified**	677	1164	942	927.67	243.82	514	810	502	608.67	174.46

Supplementary table 2. Number of dendritic spines on the examined cells.
Table showing the overall number of dendritic spines, the total number of spines on the apical and basal dendritic arbour and the number of dendritic spines from different phenotypic subtypes of the individual pyramidal cells (n = 3 infant and n = 3 late adulthood).

bioRxiv preprint doi: https://doi.org/10.1101/2024.06.13.598792; this version posted January 6, 2025. The copyright holder for this preprint (which was not certified by peer review) is the author/funder, who has granted bioRxiv a license to display the preprint in perpetuity. It is made available under aCC-BY 4.0 International license.

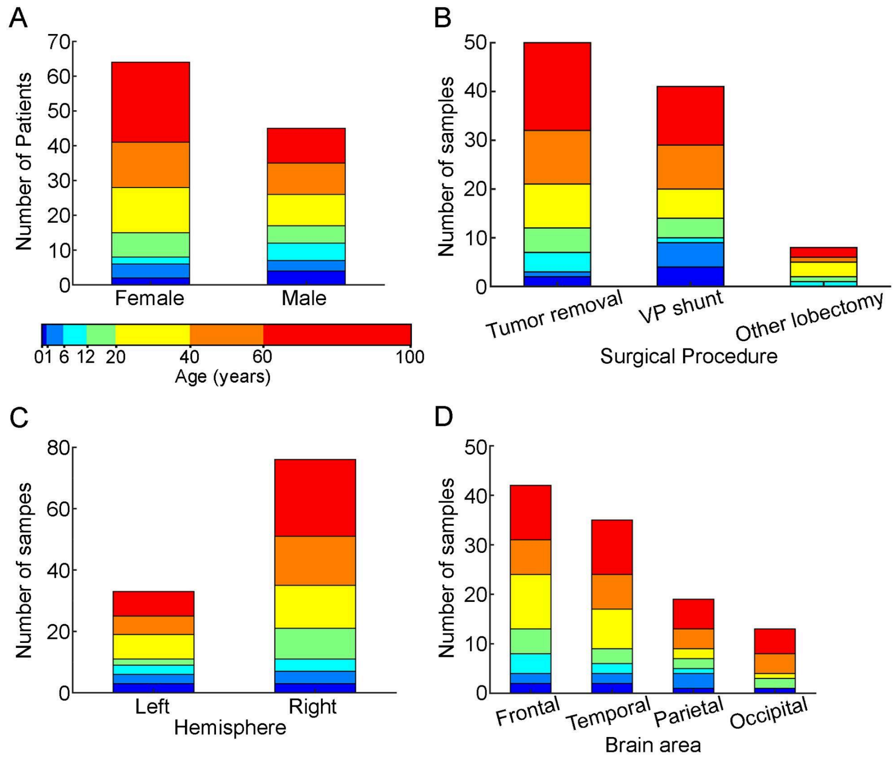

Supplementary Figure 1. Patient metadata

Distribution of patients by gender (female: infant n = 2, early childhood n = 4, late childhood n = 2, adolescence n = 7, young adulthood n = 13, middle adulthood n = 13, late adulthood n = 23, male: infant n= 4, early childhood n = 3, late childhood n = 5, adolescence n = 5, young adulthood n = 9, middle adulthood n = 9, late adulthood n = 10) (A), surgical procedure (tumor removal: infant n = 2, early childhood n = 2, late childhood n = 5, adolescence n = 7, young adulthood n = 13, middle adulthood n = 12, late adulthood n = 19, VP (ventriculoperitoneal) shunt: infant n= 4, early childhood n = 5, late childhood n = 1, adolescence n = 4, young adulthood n = 6, middle adulthood n = 9, late adulthood n = 12, other: infant n= 0, early childhood n = 0, late childhood n = 1, adolescence n = 1, young adulthood n = 3, middle adulthood n = 1, late adulthood n = 2) (B), hemisphere (left: infant n = 3, early childhood n = 3, late childhood n = 3, adolescence n = 2, young adulthood n = 8, middle adulthood n = 6, late adulthood n = 8, right: infant n= 3, early childhood n = 4, late childhood n = 4, adolescence n = 10, young adulthood n = 14, middle adulthood n = 16, late adulthood n = 25) (C) and brain area (frontal: infant n = 2, early childhood n = 2, late childhood n = 4, adolescence n = 5, young adulthood n = 11, middle adulthood n = 7, late adulthood n = 11, temporal: infant n = 2, early childhood n = 2, late childhood n = 2, adolescence n = 3, young adulthood n = 8, middle adulthood n = 7, late adulthood n = 11, parietal: infant n = 1, early childhood n = 3, late childhood n = 1, adolescence n = 2, young adulthood n = 2, middle adulthood n = 4, late adulthood n = 6, occipital: infant n = 1, early childhood n = 0, late childhood n = 0, adolescence n = 2, young adulthood n = 1, middle adulthood n = 4, late adulthood n = 5)(D). Stacked columns are colored regarding the age groups shown on the colorbar.

bioRxiv preprint doi: https://doi.org/10.1101/2024.06.13.598792; this version posted January 6, 2025. The copyright holder for this preprint (which was not certified by peer review) is the author/funder, who has granted bioRxiv a license to display the preprint in perpetuity. It is made available under aCC-BY 4.0 International license.

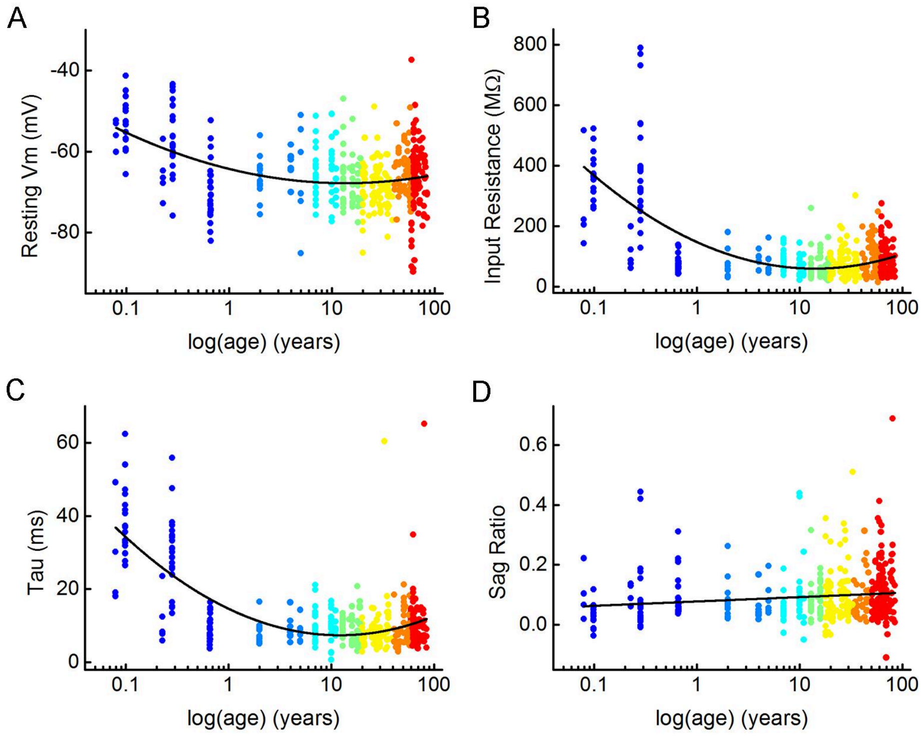

Supplementary Figure 2. Distribution of subthreshold electrophysiological features with age. (A-D) Scatter plots showing the passive electrophysiological characteristics: resting membrane potential (A), input resistance (B), tau (C), and sag ratio (D) throughout the lifespan. Dots are colored according to the age groups, age is represented in years on a logarithmic scale.

bioRxiv preprint doi: https://doi.org/10.1101/2024.06.13.598792; this version posted January 6, 2025. The copyright holder for this preprint (which was not certified by peer review) is the author/funder, who has granted bioRxiv a license to display the preprint in perpetuity. It is made available under aCC-BY 4.0 International license.

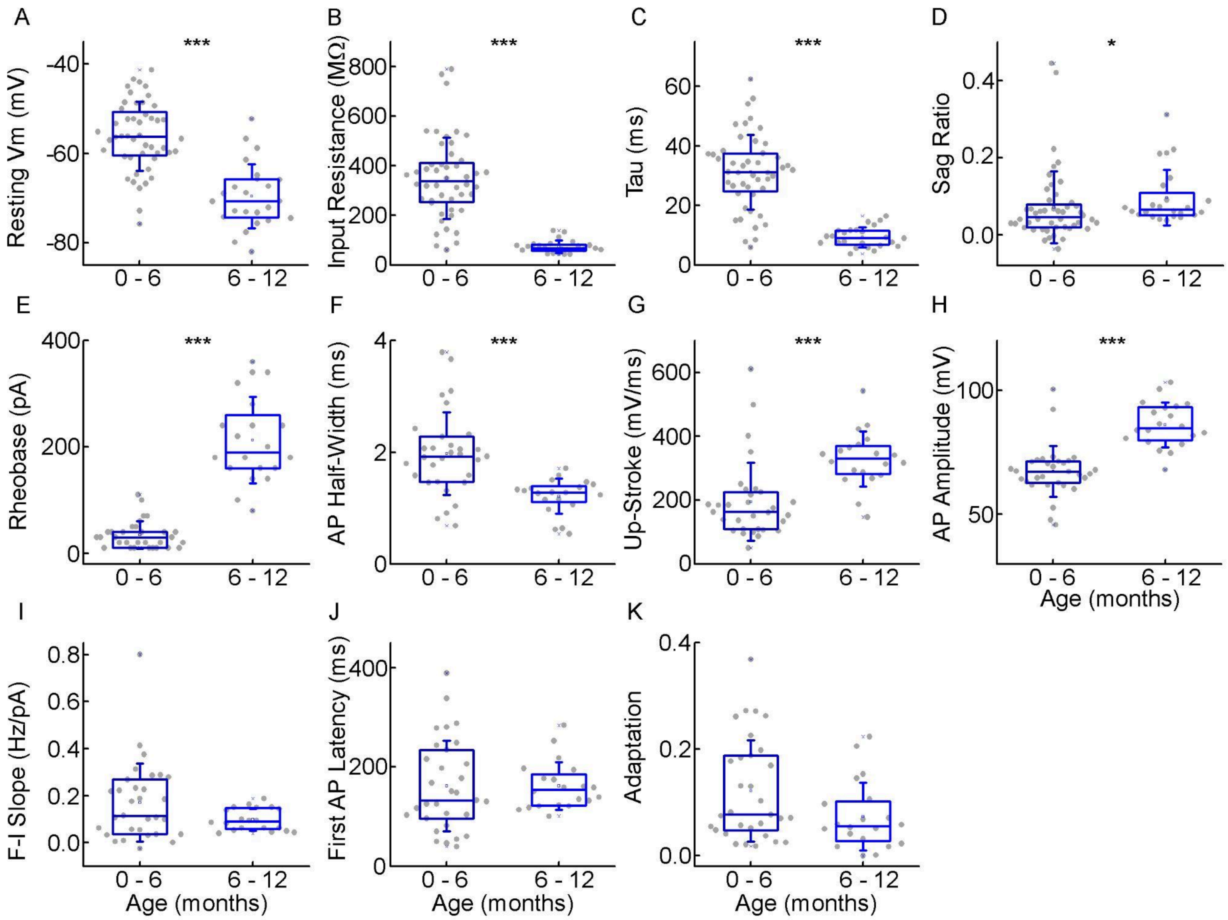

Supplementary Figure 3. Electrophysiological differences during the first year of life
A-D, Boxplots showing differences in passive properties, resting membrane potential (A), input resistance (B), tau (C) and sag ratio (D) within the infant age group (* P < 0.05, ** P < 0.01, *** P < 0.001, two sample t-test (A, B, C) or Mann-Whitney test (D)).
E-H, Differences in the action potential kinetics, Rheobase (E), AP half-width (F), up-stroke (G), and AP amplitude (H) between the cells from the first and the second half of the first year of life. Asterisks indicate significance (* P < 0.05, ** P < 0.01, *** P < 0.001, Mann-Whitney test).
I-K, Deviations in AP firing pattern parameters, F-I slope (H), first AP latency (I), and adaptation (J) during infancy.

bioRxiv preprint doi: https://doi.org/10.1101/2024.06.13.598792; this version posted January 6, 2025. The copyright holder for this preprint (which was not certified by peer review) is the author/funder, who has granted bioRxiv a license to display the preprint in perpetuity. It is made available under aCC-BY 4.0 International license.

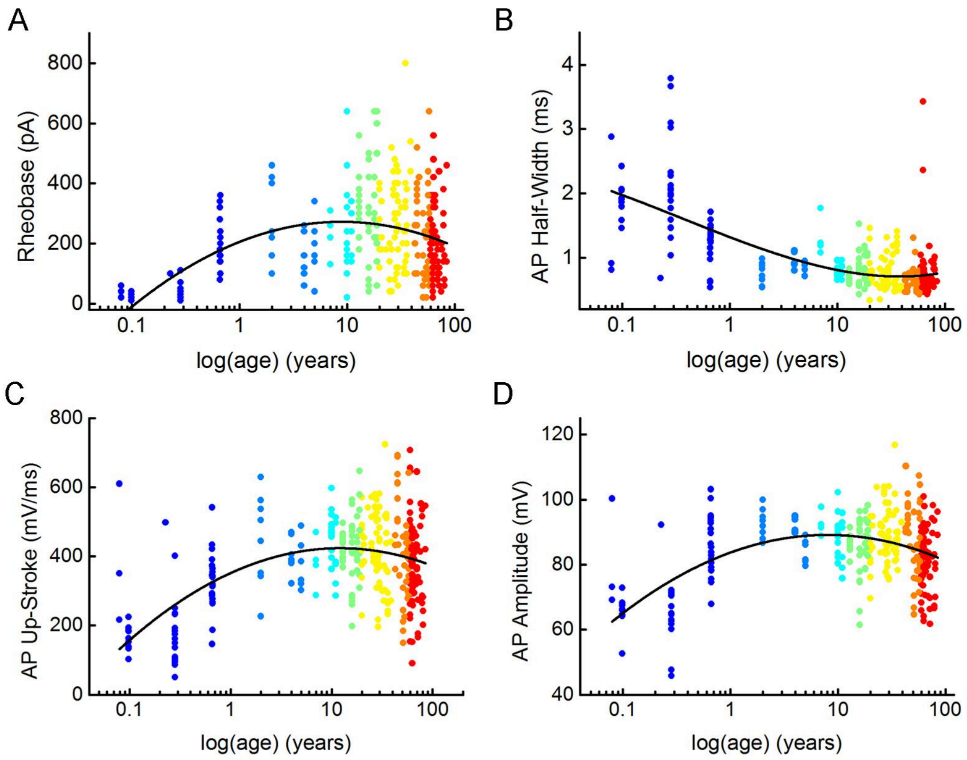

Supplementary figure 4. Distribution of suprathreshold electrophysiological features with age. (A-D) Diagrams show changes through age in rheobase (A), action potential half-width (B), action potential up-stroke (C), and action potential amplitude (D). Dots are colored according to the age groups, age is represented in years on a logarithmic scale.

bioRxiv preprint doi: https://doi.org/10.1101/2024.06.13.598792; this version posted January 6, 2025. The copyright holder for this preprint (which was not certified by peer review) is the author/funder, who has granted bioRxiv a license to display the preprint in perpetuity. It is made available under aCC-BY 4.0 International license.

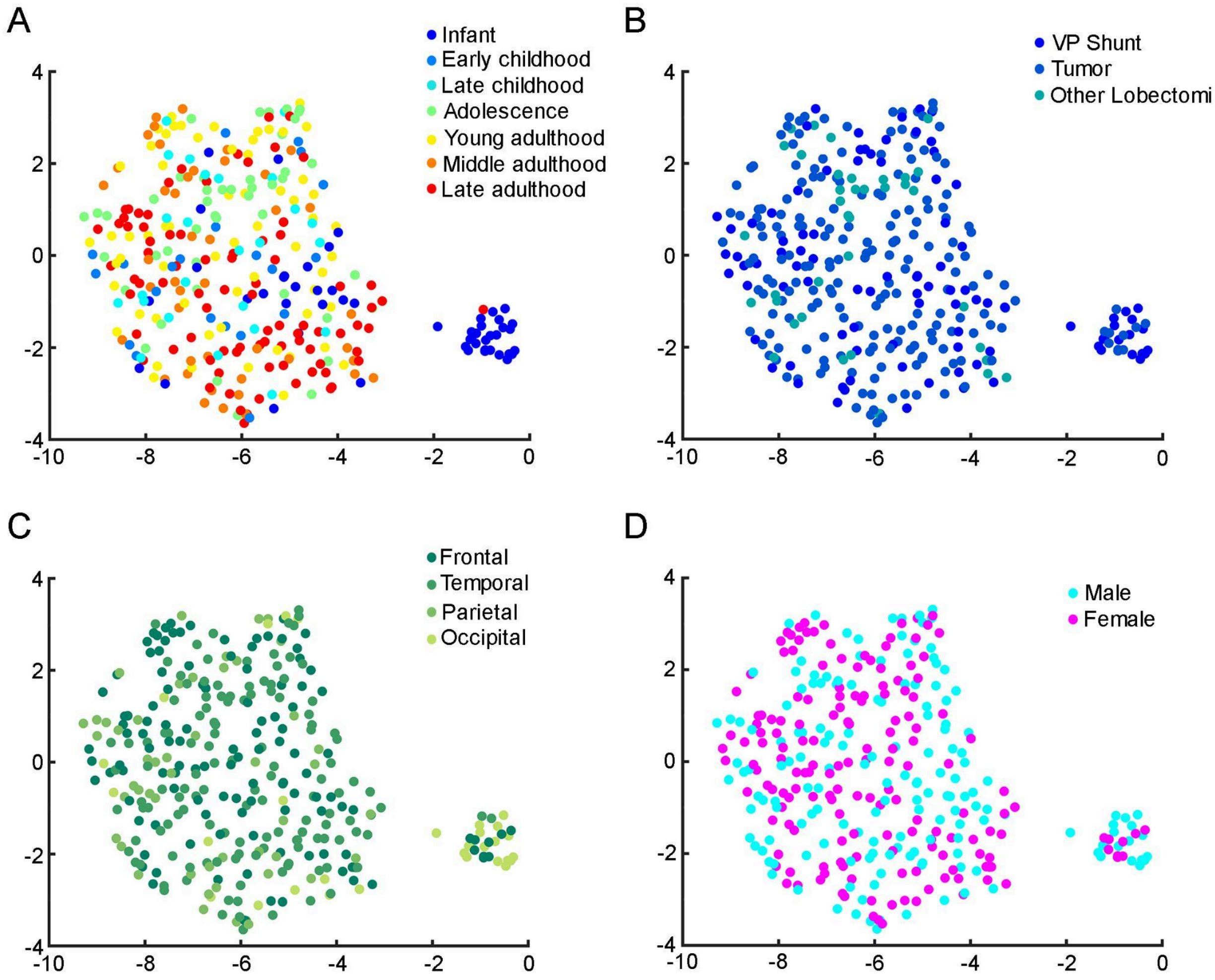

Supplementary Figure 5. Relationship between patient metadata and electrophysiology. Uniform Manifold Approximation and Projection (UMAP) of 8 electrophysiological properties (resting Vm, input resistance, tau, sag ratio, rheobase, AP half-width, AP up-stroke, and AP amplitude) with data points for 331 cortical L2/3 pyramidal cells, colored with the corresponding age groups (A), surgical procedures (B), brain area (C) and gender (D).

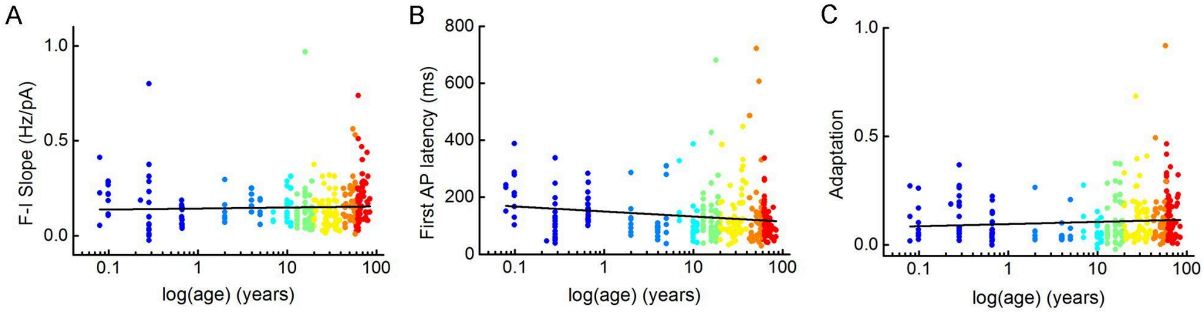

Supplementary Figure 6. Distribution of firing pattern characteristics with age.

bioRxiv preprint doi: https://doi.org/10.1101/2024.06.13.598792; this version posted January 6, 2025. The copyright holder for this preprint (which was not certified by peer review) is the author/funder, who has granted bioRxiv a license to display the preprint in perpetuity. It is made available under aCC-BY 4.0 International license.

(A-C) Plots show F-I slope (A), first AP latency (B), and adaptation of APs (C) depending on age. Dots are colored according to the age groups, age is represented in years on a logarithmic scale.

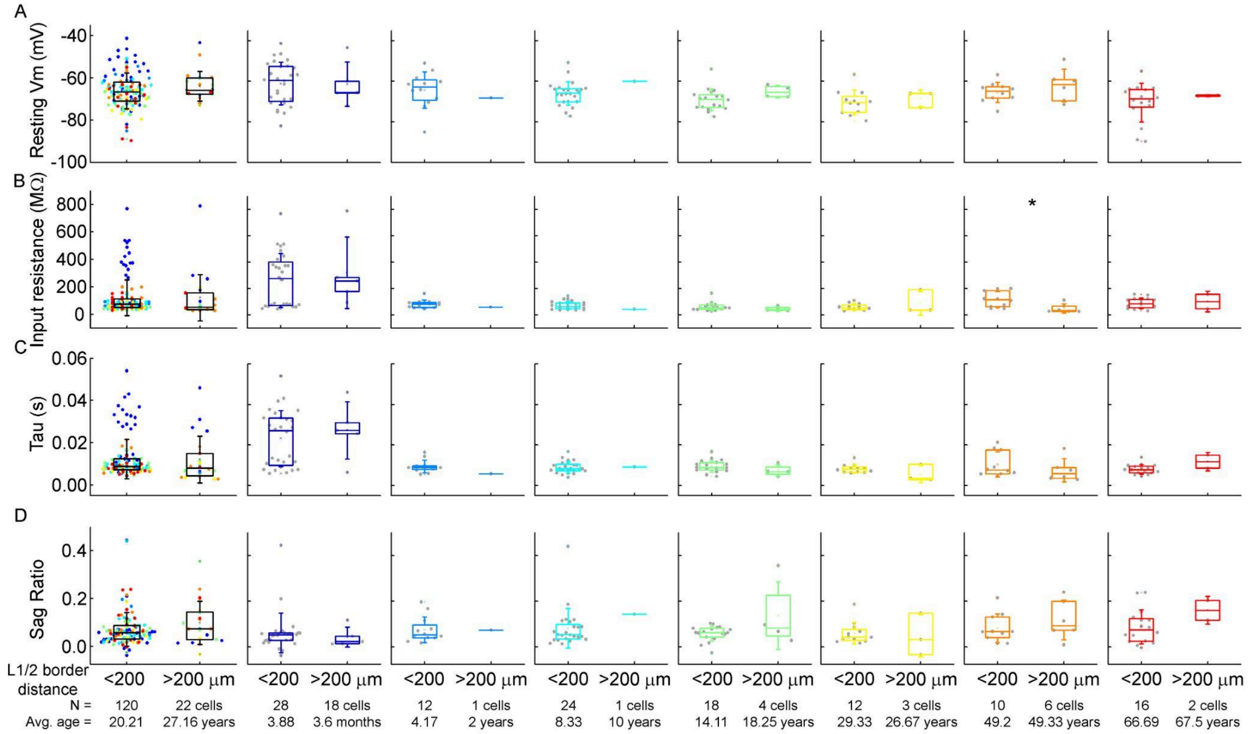

Supplementary Figure 7. Comparison of the subthreshold properties of the cells as a function of their distance from the layer border.
A-D: Box plots show resting membrane potential (A), input resistance (B), tau (C), and sag ratio (D) of the pyramidal cells whose soma is located at a distance greater than and less than 200 µm from the L1 border. From left to right: data from all age groups, infant, early childhood, late childhood, adolescence, young adulthood, middle adulthood and late adulthood groups. Asterisks indicate significance (* $P < 0.05$, Mann-Whitney test).

bioRxiv preprint doi: https://doi.org/10.1101/2024.06.13.598792; this version posted January 6, 2025. The copyright holder for this preprint (which was not certified by peer review) is the author/funder, who has granted bioRxiv a license to display the preprint in perpetuity. It is made available under aCC-BY 4.0 International license.

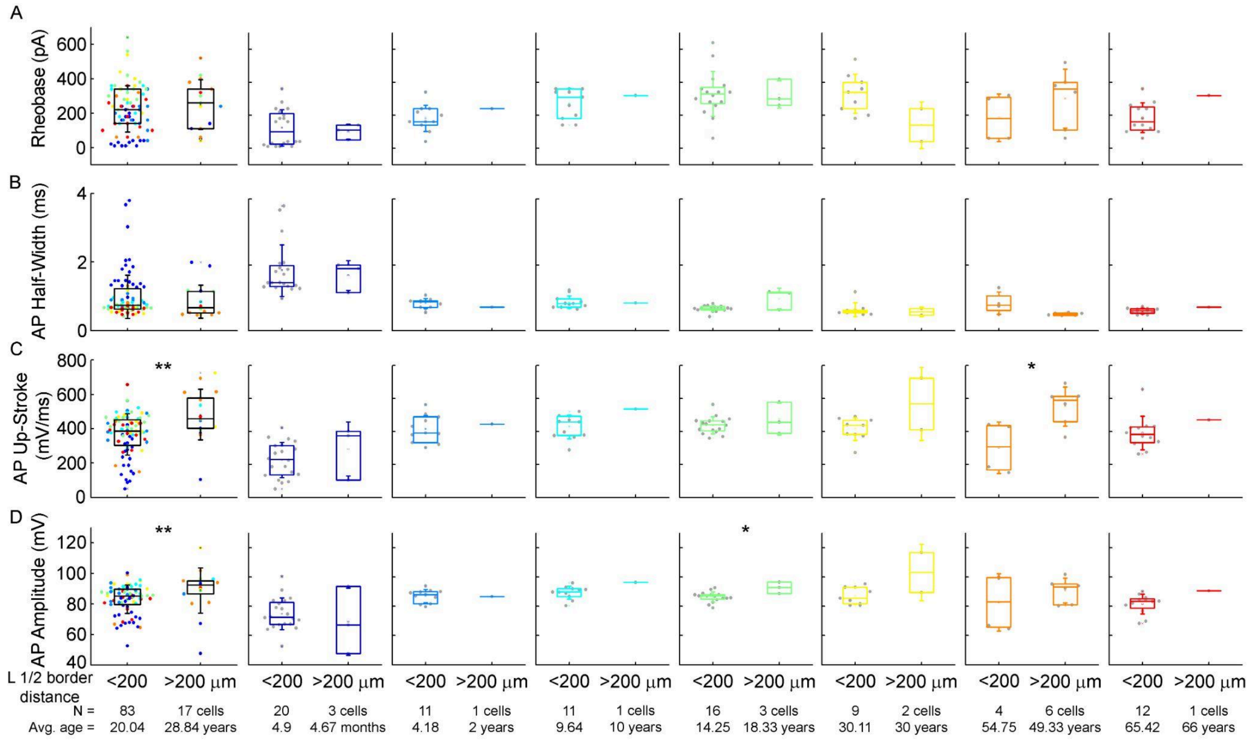

Supplementary Figure 8. Comparison of the action potential properties of the cells as a function of their distance from the layer border.

A-C: Boxplots show rheobase (A), action potential half-width (B), action potential upstroke (C) and action potential amplitude (D) of cells whose soma is located at a distance greater than and less than 200 µm from the L1 border. From left to right: data from all age groups, infant, early childhood, late childhood, adolescence, young adulthood, middle adulthood and late adulthood. Asterisks indicate significance (* P < 0.05, ** P < 0.01, Mann-Whitney test).

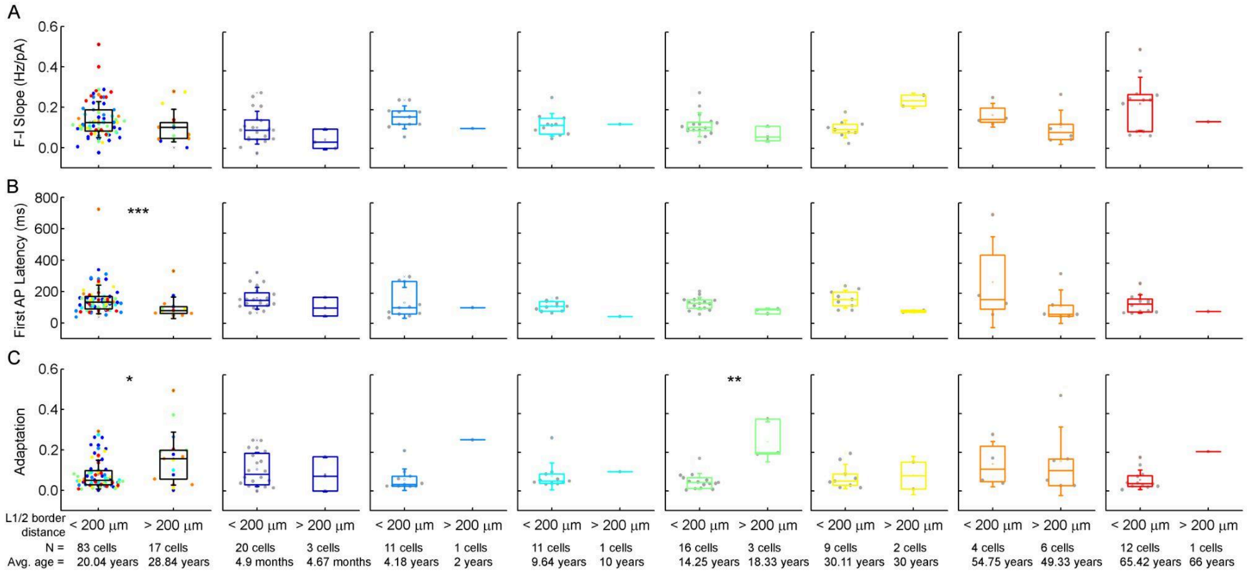

bioRxiv preprint doi: https://doi.org/10.1101/2024.06.13.598792; this version posted January 6, 2025. The copyright holder for this preprint (which was not certified by peer review) is the author/funder, who has granted bioRxiv a license to display the preprint in perpetuity. It is made available under aCC-BY 4.0 International license.

Supplementary Figure 9. Comparison of the firing pattern characteristics of the cells as a function of their distance from the layer border.
A-C: Boxplots show: F-I slope (A), latency of the first AP (B) and adaptation of APs (C) of cells whose soma is located at a distance greater than and less than 200 µm from the L1 border. From left to right: data from all ages, infancy, early childhood, late childhood, adolescence, young adulthood, middle adulthood and late adulthood. Asterisks indicate significance (* $P < 0.05$, ** $P < 0.01$, Mann-Whitney test).

bioRxiv preprint doi: https://doi.org/10.1101/2024.06.13.598792; this version posted January 6, 2025. The copyright holder for this preprint (which was not certified by peer review) is the author/funder, who has granted bioRxiv a license to display the preprint in perpetuity. It is made available under aCC-BY 4.0 International license.

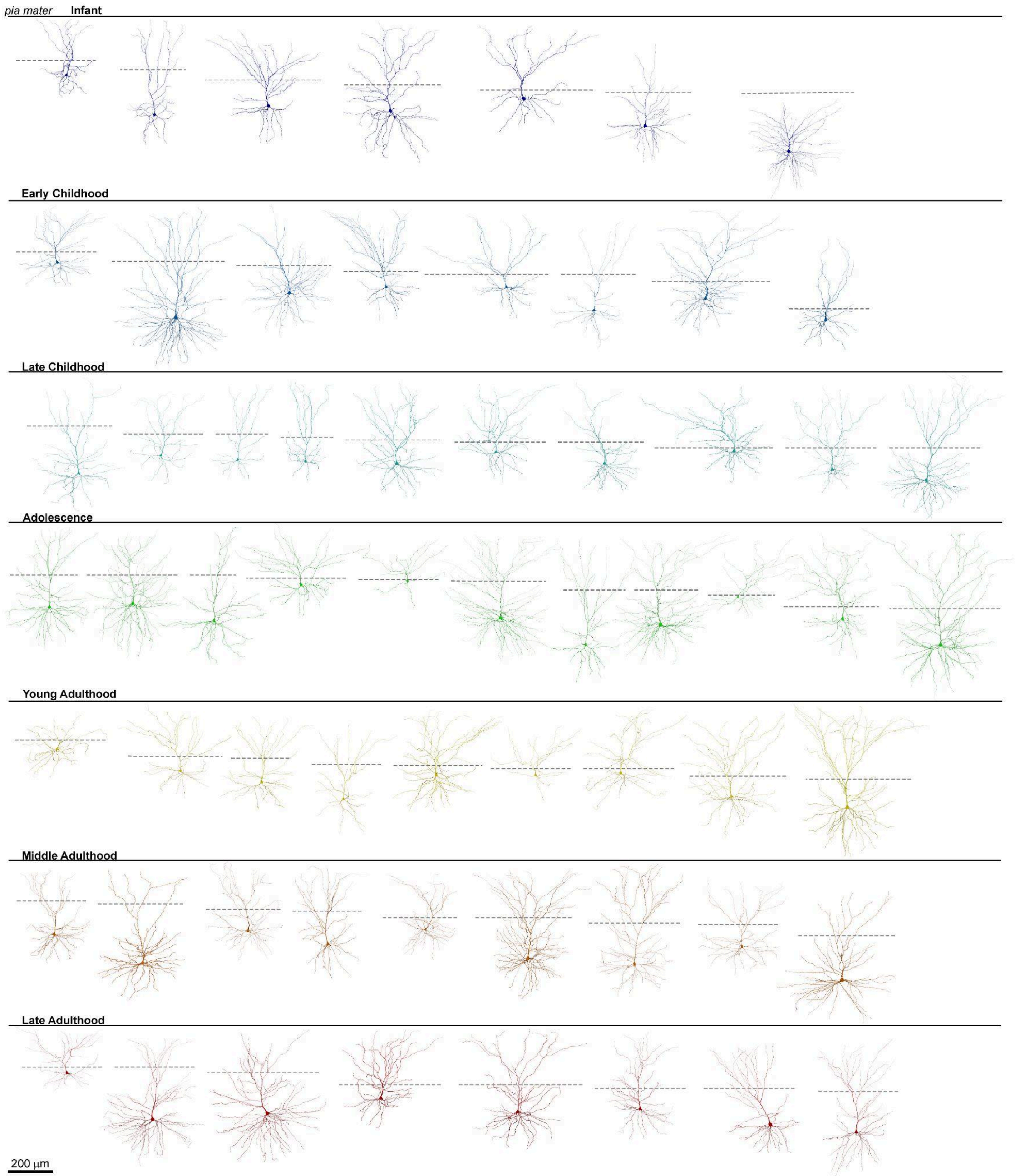

Supplementary Figure 10. Human cortical L2/3 pyramidal cell dendritic reconstructions.

bioRxiv preprint doi: https://doi.org/10.1101/2024.06.13.598792; this version posted January 6, 2025. The copyright holder for this preprint (which was not certified by peer review) is the author/funder, who has granted bioRxiv a license to display the preprint in perpetuity. It is made available under aCC-BY 4.0 International license.

Reconstructions (n = 63) of the examined human cortical pyramidal cells, from top to bottom: infant, early childhood, late childhood, adolescence, young adulthood, middle adulthood and late adulthood age groups. Black lines are representing the pia mater, gray dashed lines represent the L1-L2 border.

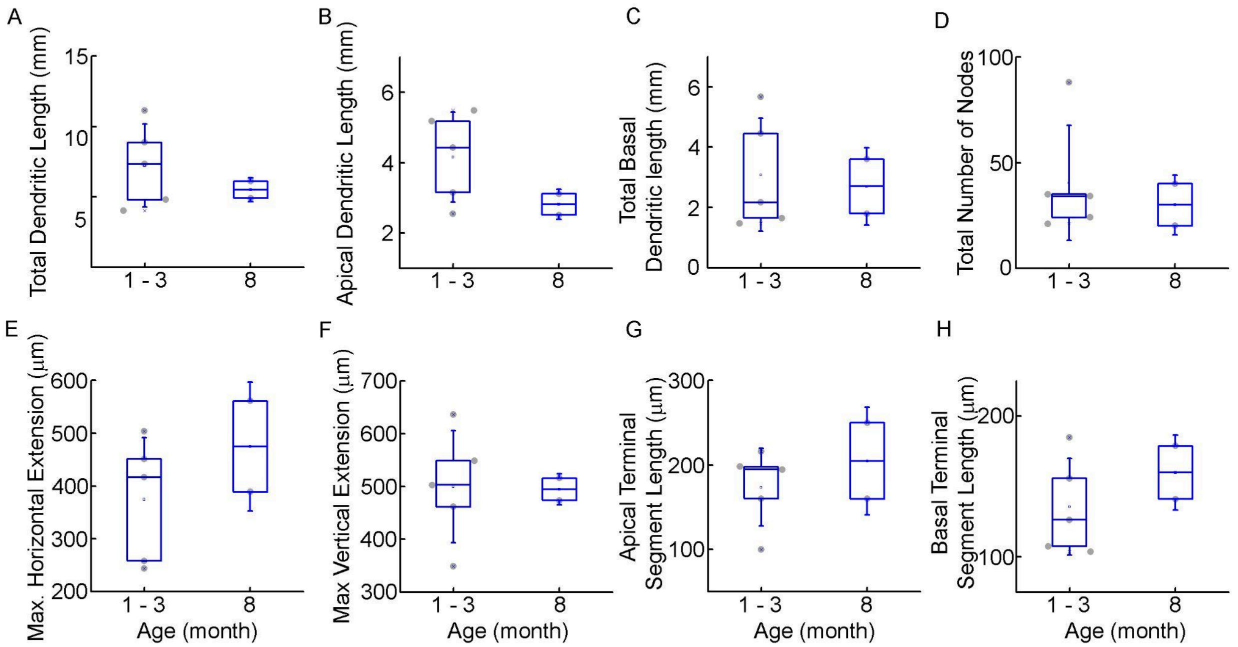

Supplementary Figure 11. Morphological comparison of the examined infant cells.
A-H: Boxplots show morphological features: total (A), apical (B), total basal (C) dendritic length, total number of nodes (D), maximal horizontal (E), and vertical (F) extension, average apical (G) and basal (H) terminal dendritic segment from infant patient during the first and second half of the first year of life.

bioRxiv preprint doi: https://doi.org/10.1101/2024.06.13.598792; this version posted January 6, 2025. The copyright holder for this preprint (which was not certified by peer review) is the author/funder, who has granted bioRxiv a license to display the preprint in perpetuity. It is made available under aCC-BY 4.0 International license.

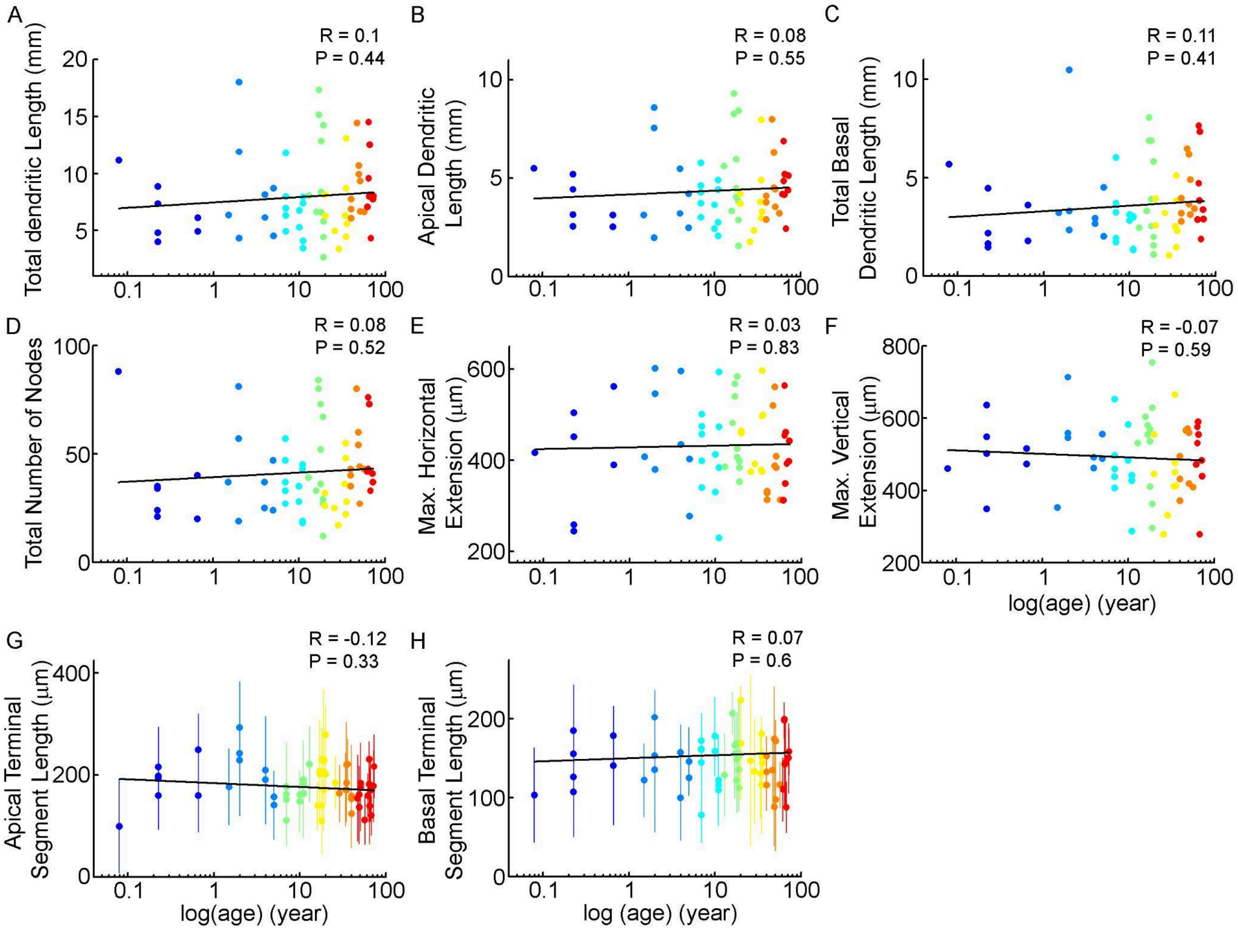

Supplementary Figure 12. Distribution of morphological features with age

A-F: Scatterplots show the distribution of total (A), apical (B), total basal dendritic length (C), total number of bifurcations (D), maximum horizontal extension (E), maximum vertical extension of the dendritic arborisation (F). The age is represented in years on a logarithmic scale.

G-H: Scatterplots show the average apical (G) and basal (H) terminal segment length of the cells, dots indicate the mean segment length of cells, vertical lines show the standard deviation.

bioRxiv preprint doi: https://doi.org/10.1101/2024.06.13.598792; this version posted January 6, 2025. The copyright holder for this preprint (which was not certified by peer review) is the author/funder, who has granted bioRxiv a license to display the preprint in perpetuity. It is made available under aCC-BY 4.0 International license.

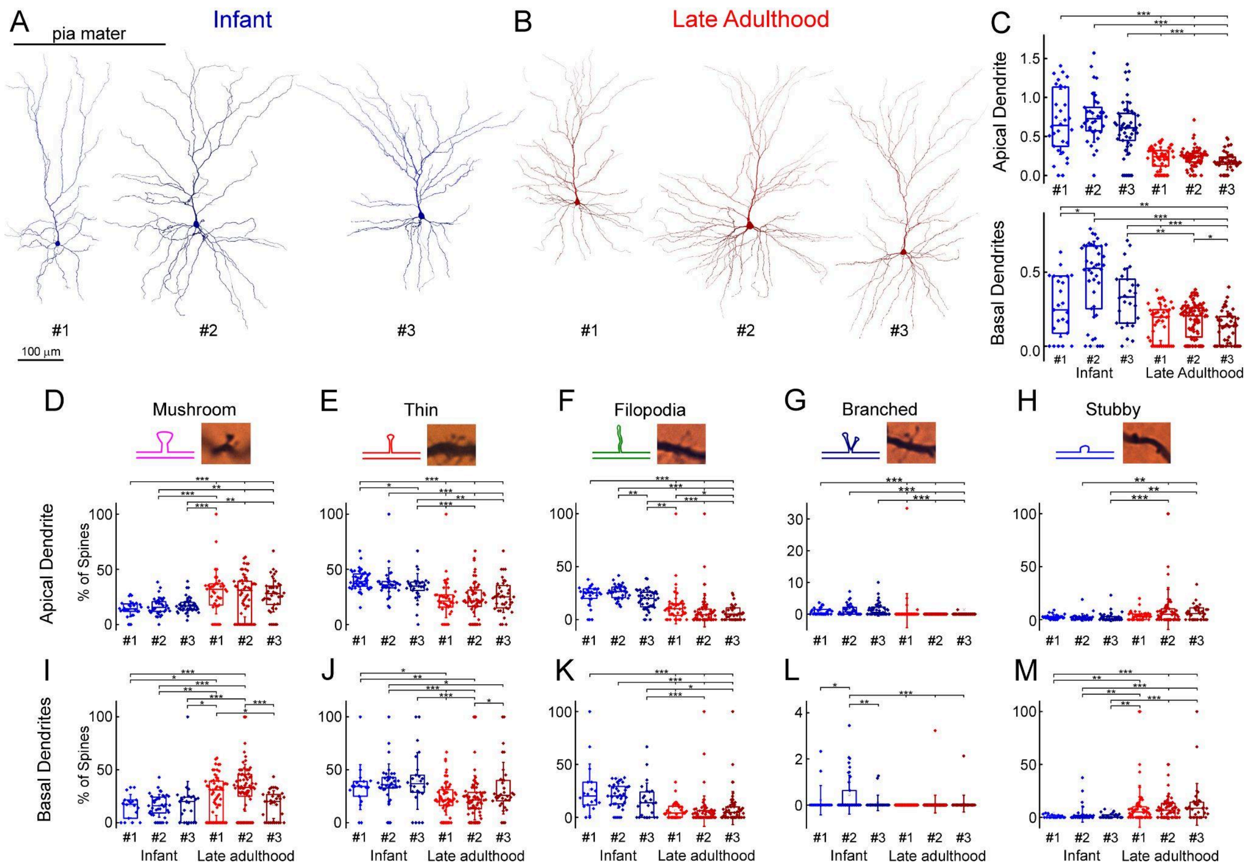

Supplementary Figure 13.

(A) Anatomical 3D reconstructions of the examined n = 3 human L2/3 pyramidal cells from the infant age group.

(B) Anatomical reconstructions (n = 3) from the late adulthood group.

(C) Boxplots showing the spine density on the six individual cells, the infant pyramidal cell shown with blue, the late adulthood pyramidal cell with red (cells are numbered according to figure A, B), on the apical (top) and the basal (bottom) dendrites. Asterisks indicate significance (* $P < 0.05$, ** $P < 0.01$, *** $P < 0.001$, Kruskal-Wallis test with post-hoc Dunn test).

(D-H) The plots show the distribution of mushroom (D), thin (E), filopodium (F), branched (G), and stubby (H) dendritic spine types on the apical dendrites of the reconstructed infant (n = 3, blue) and late adult (n = 3, red) pyramidal cells. Top, schematic illustration and representative images representation of the examined dendritic spine types. Bottom, spine distributions on the individual cells that were examined. Asterisks indicate significance (* $P < 0.05$, ** $P < 0.01$, *** $P < 0.001$, Kruskal-Wallis test with post-hoc Dunn test).

(I-M) Same as D-H but on basal dendrites.

bioRxiv preprint doi: https://doi.org/10.1101/2024.06.13.598792; this version posted January 6, 2025. The copyright holder for this preprint (which was not certified by peer review) is the author/funder, who has granted bioRxiv a license to display the preprint in perpetuity. It is made available under aCC-BY 4.0 International license.

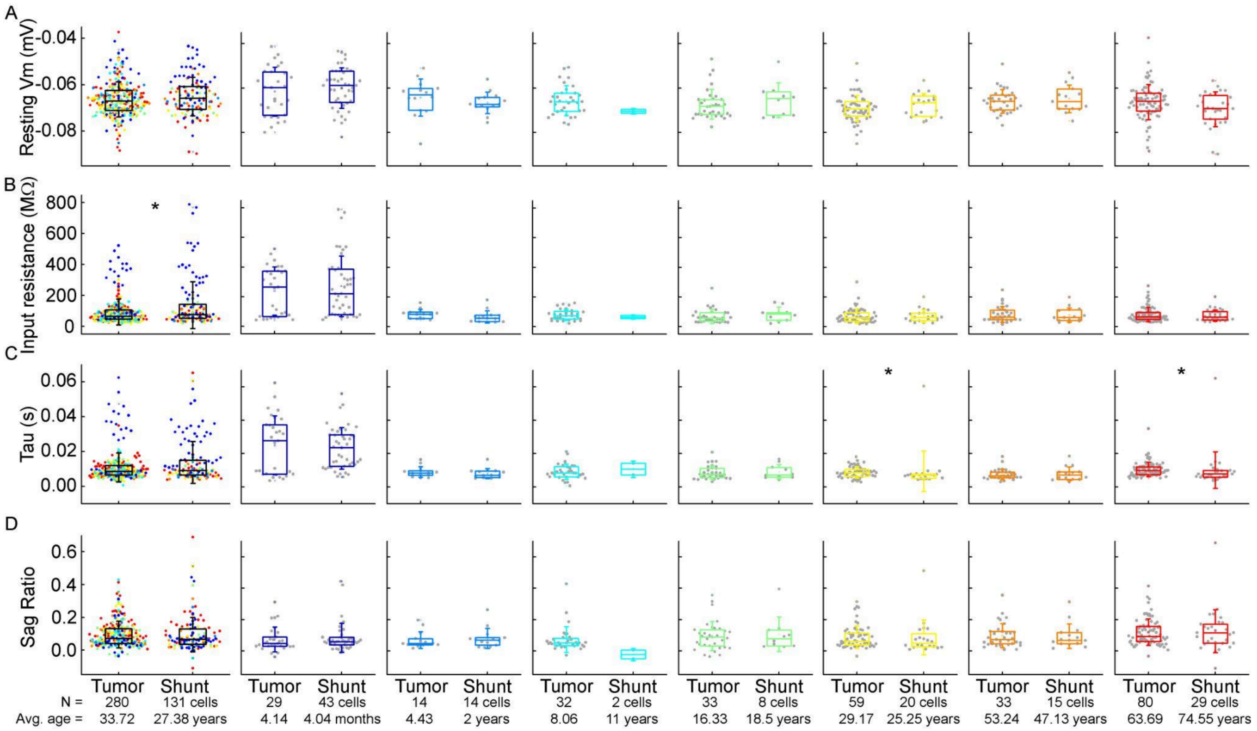

Supplementary Figure 14. Comparison of subthreshold electrophysiological properties of the examined cells from patients with tumor removal or VP shunt surgical procedures.
A-D: Box plots show resting membrane potential (A), input resistance (B), tau (C), and sag ratio (D) from patients with tumor removal (tumor) or VP shunt (shunt) surgical procedures. From left to right: data from all age groups, infant, early childhood, late childhood, adolescence, young adulthood, middle adulthood and late adulthood groups. Asterisks indicate significance (* P < 0.05, Mann-Whitney test).

bioRxiv preprint doi: https://doi.org/10.1101/2024.06.13.598792; this version posted January 6, 2025. The copyright holder for this preprint (which was not certified by peer review) is the author/funder, who has granted bioRxiv a license to display the preprint in perpetuity. It is made available under aCC-BY 4.0 International license.

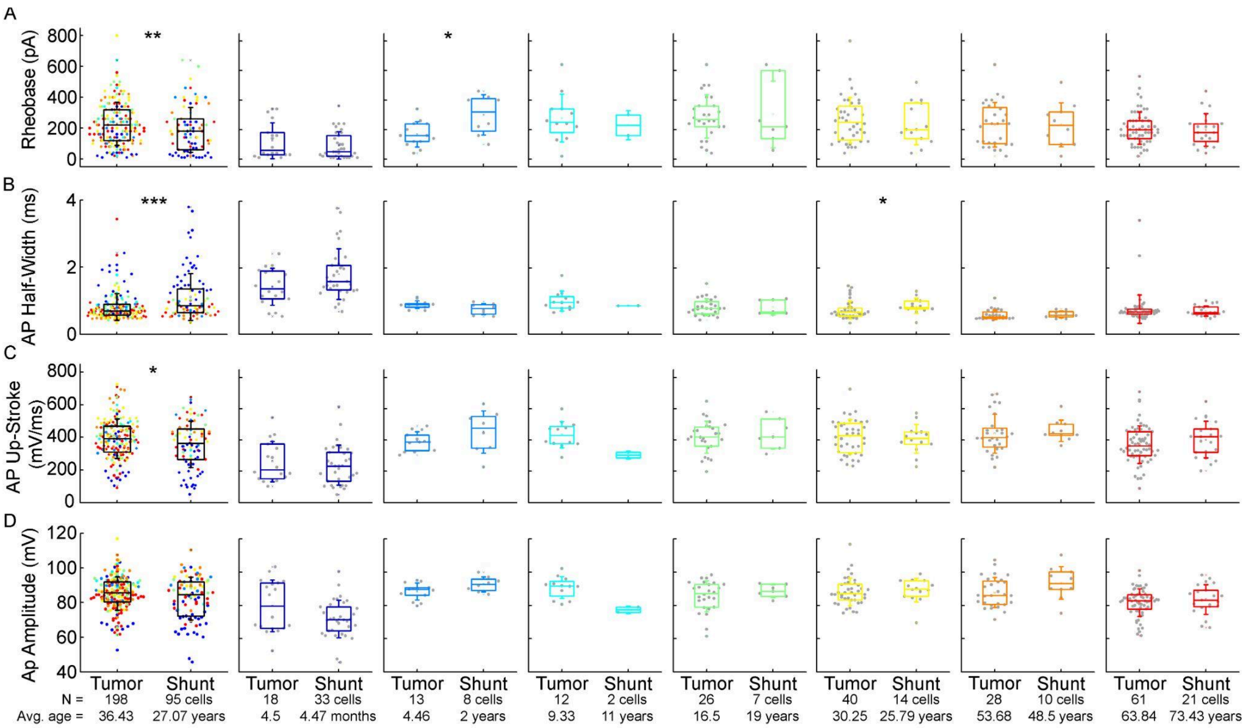

Supplementary Figure 15. Comparison of action potential properties of the examined cells from patients with tumor removal or VP shunt surgical procedures.

A-D: Boxplots show rheobase (A), action potential half-width mean (B), action potential up-stroke (C) and action potential amplitude (D) from patients with tumor removal (tumor) or VP shunt (shunt) surgical procedures. From left to right: data from all age groups, infant, early childhood, late childhood, adolescence, young adulthood, middle adulthood and late adulthood groups. Asterisks indicate significance (* P < 0.05, ** P < 0.01, *** P < 0.001, Mann-Whitney test).

bioRxiv preprint doi: https://doi.org/10.1101/2024.06.13.598792; this version posted January 6, 2025. The copyright holder for this preprint (which was not certified by peer review) is the author/funder, who has granted bioRxiv a license to display the preprint in perpetuity. It is made available under aCC-BY 4.0 International license.

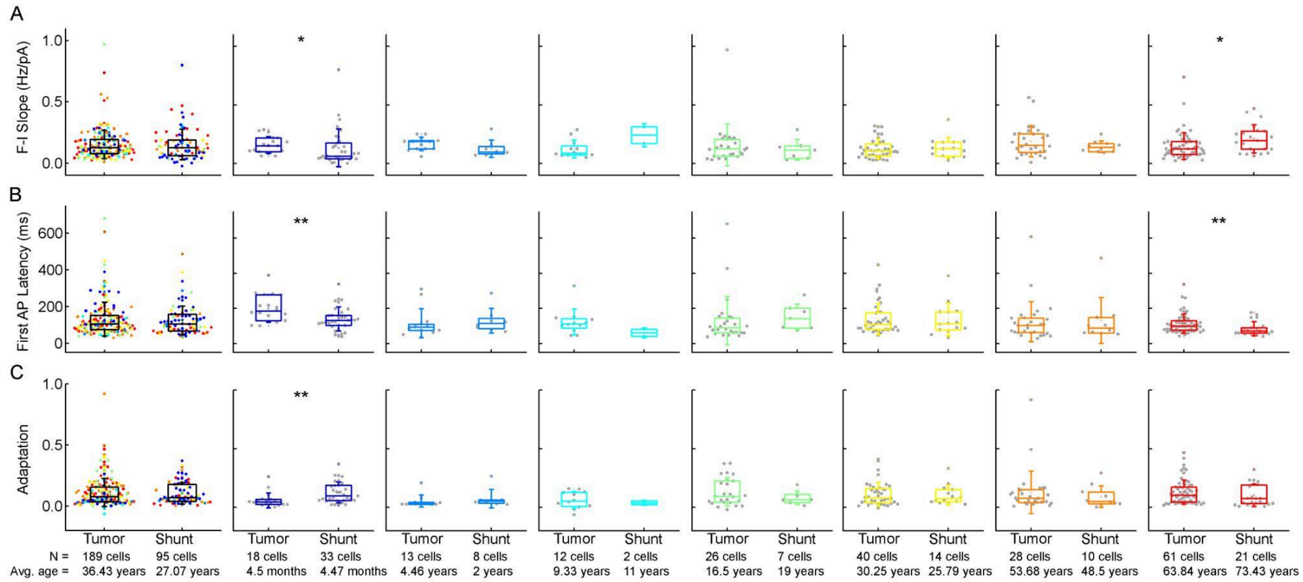

Supplementary Figure 16. Comparison of firing pattern characteristics of the examined cells from patients with tumor removal or VP shunt surgical procedures.

A-C: Box plots showing: F-I slope (A), first AP latency (B), and adaptation of APs (C) from patients with tumor removal (tumor) or VP shunt (shunt) surgical procedures. From left to right: data from all age groups, infant, early childhood, late childhood, adolescence, young adulthood, middle adulthood and late adulthood groups. Asterisks indicate significance (* P < 0.05, ** P < 0.01, Mann-Whitney test).

bioRxiv preprint doi: https://doi.org/10.1101/2024.06.13.598792; this version posted January 6, 2025. The copyright holder for this preprint (which was not certified by peer review) is the author/funder, who has granted bioRxiv a license to display the preprint in perpetuity. It is made available under aCC-BY 4.0 International license.

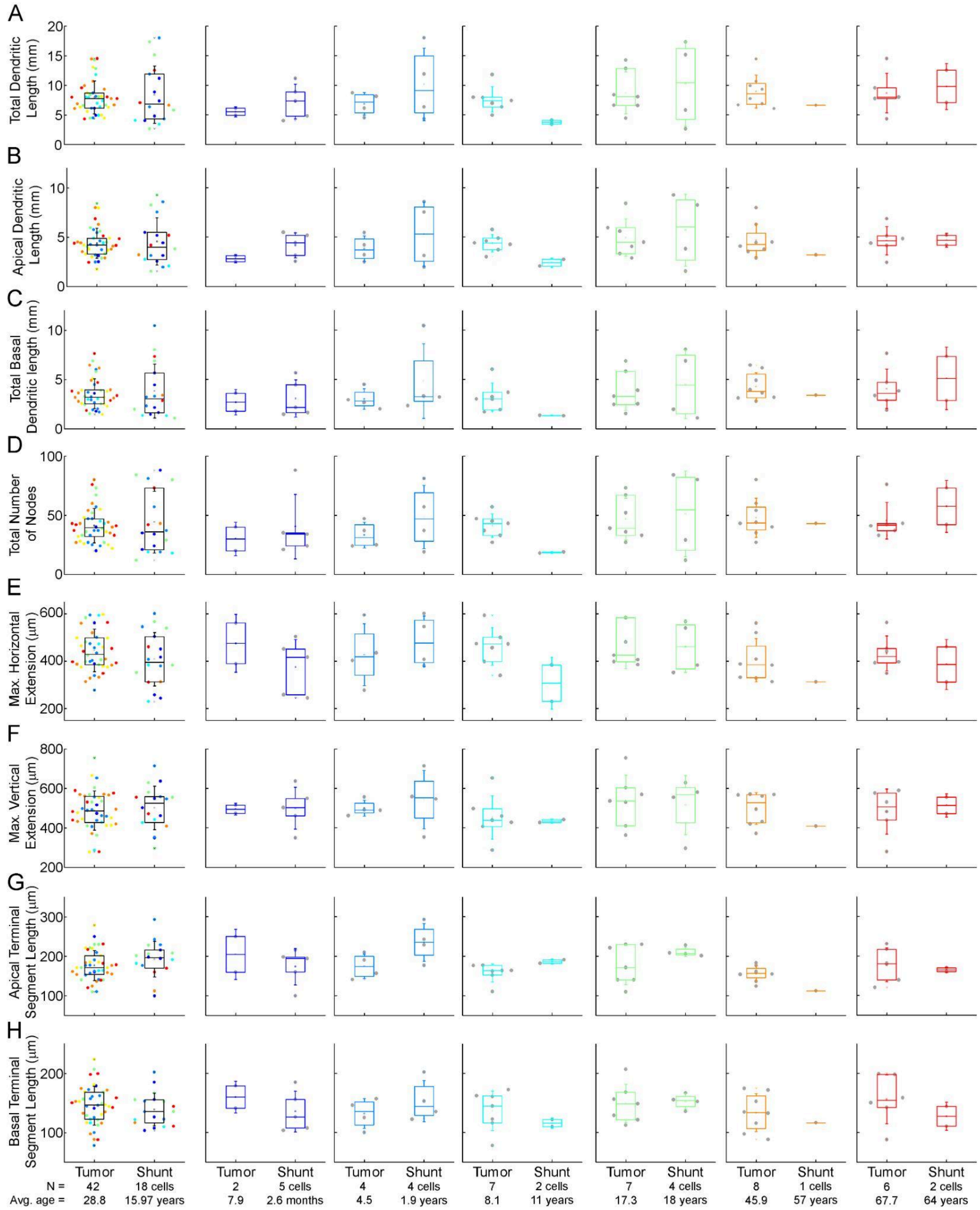

Supplementary Figure 17. Morphological comparison of the examined cells from patients with tumor removal or VP shunt surgical procedures.

A-H: Boxplots showing morphological features: total (A), apical (B), total basal (C), dendritic length, total number of nodes (D), maximal horizontal (E), and vertical (F) extension, average

bioRxiv preprint doi: https://doi.org/10.1101/2024.06.13.598792; this version posted January 6, 2025. The copyright holder for this preprint (which was not certified by peer review) is the author/funder, who has granted bioRxiv a license to display the preprint in perpetuity. It is made available under aCC-BY 4.0 International license.

apical (G) and basal (H) terminal dendritic segment from patients with tumor removal (tumor) or VP shunt (shunt) surgical procedures. From left to right: data from all age groups, infant, early childhood, late childhood, adolescence, middle adulthood and late adulthood groups. Asterisks indicate significance (* P < 0.05, Mann-Whitney test).

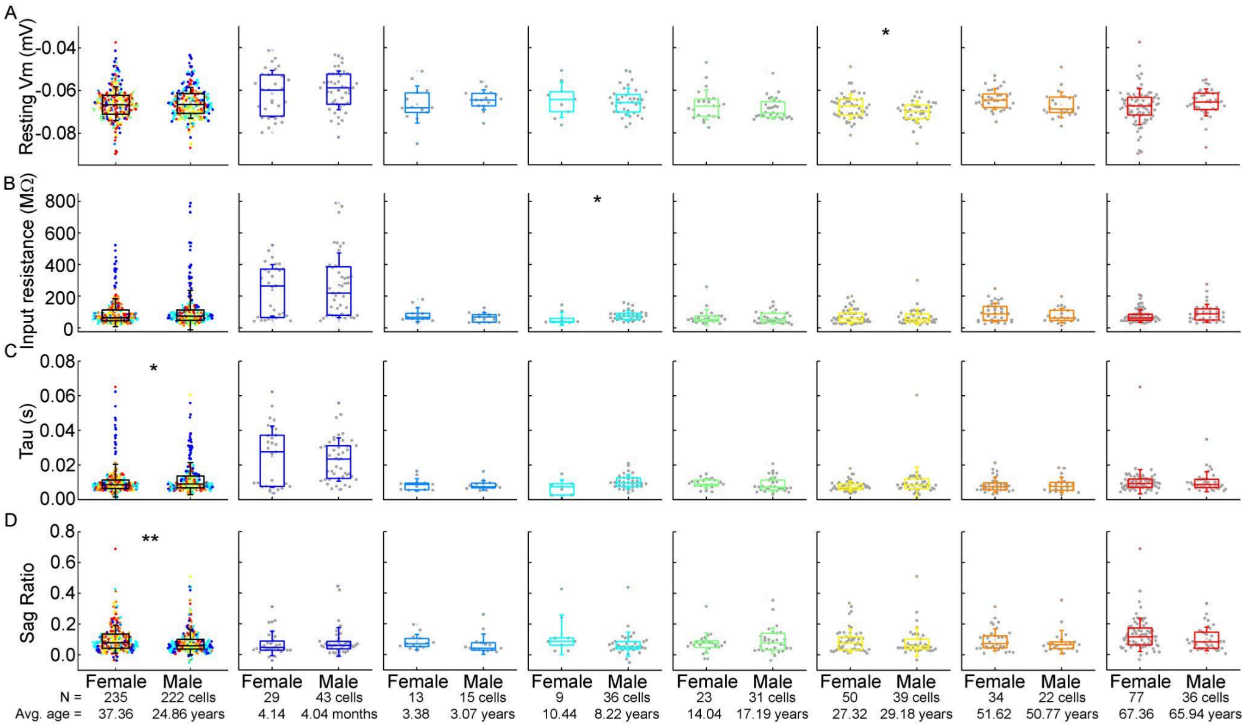

Supplementary Figure 18. Comparison of subthreshold electrophysiological properties of the examined cells from female and male patients.

A-D: Box plots show resting membrane potential (A), input resistance (B), tau (C), and sag ratio (D) from female and male patients. From left to right: data from all age groups, infant, early childhood, late childhood, adolescence, young adulthood, middle adulthood and late adulthood groups. Asterisks indicate significance (* P < 0.05, ** P < 0.01 Mann-Whitney test or two sample t-test).

bioRxiv preprint doi: https://doi.org/10.1101/2024.06.13.598792; this version posted January 6, 2025. The copyright holder for this preprint (which was not certified by peer review) is the author/funder, who has granted bioRxiv a license to display the preprint in perpetuity. It is made available under aCC-BY 4.0 International license.

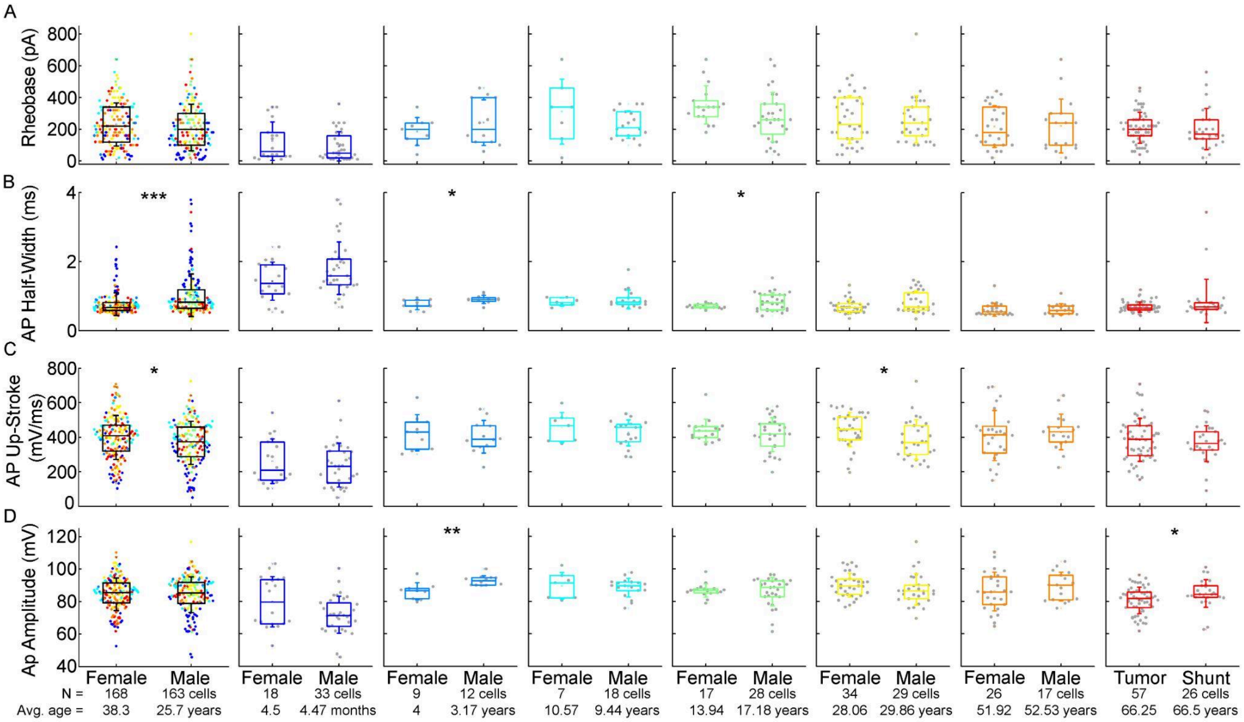

Supplementary Figure 19. Comparison of action potential properties of the examined cells from female and male patients.
A-D: Boxplots show rheobase (A), action potential half-width mean (B), action potential up-stroke (C) and action potential amplitude (D) from female and male patients. From left to right: data from all age groups, infant, early childhood, late childhood, adolescence, young adulthood, middle adulthood and late adulthood groups. Asterisks indicate significance (* P < 0.05, ** P < 0.01, *** P < 0.001, Mann-Whitney test or two sample t-test).

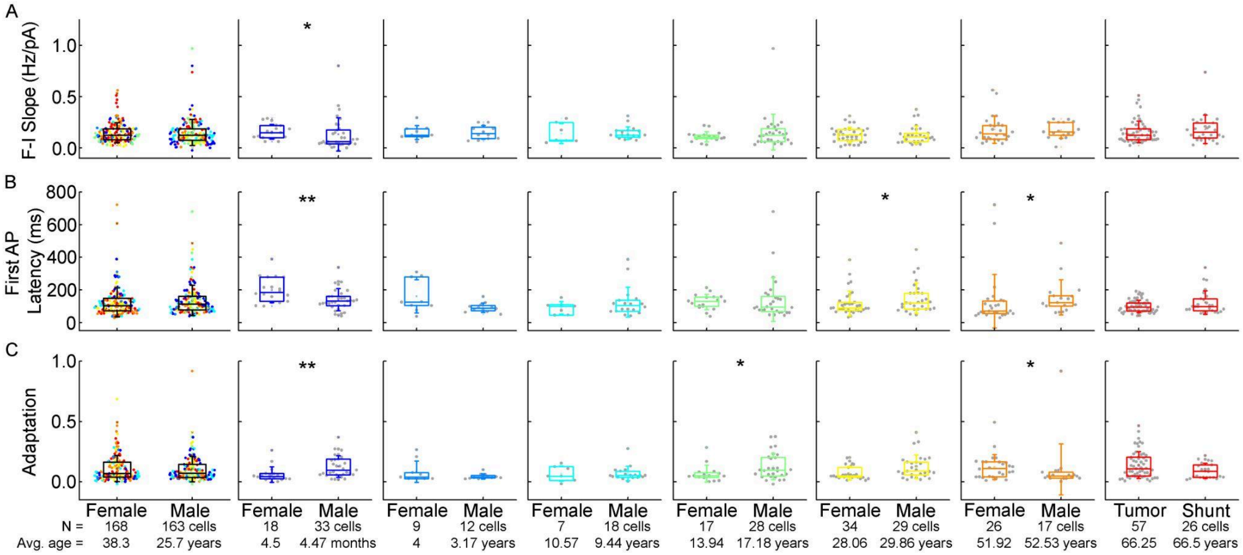

Supplementary Figure 20. Comparison of firing pattern characteristics of the examined cells from female and male patients.

bioRxiv preprint doi: https://doi.org/10.1101/2024.06.13.598792; this version posted January 6, 2025. The copyright holder for this preprint (which was not certified by peer review) is the author/funder, who has granted bioRxiv a license to display the preprint in perpetuity. It is made available under aCC-BY 4.0 International license.

A-C: Box plots showing: F-I slope (A), first AP latency (B), and adaptation of APs (C) from female and male patients. From left to right: data from all age groups, infant, early childhood, late childhood, adolescence, young adulthood, middle adulthood, and late adulthood groups. Asterisks indicate significance (* P < 0.05, ** P < 0.01, Mann-Whitney test or two sample t-test).

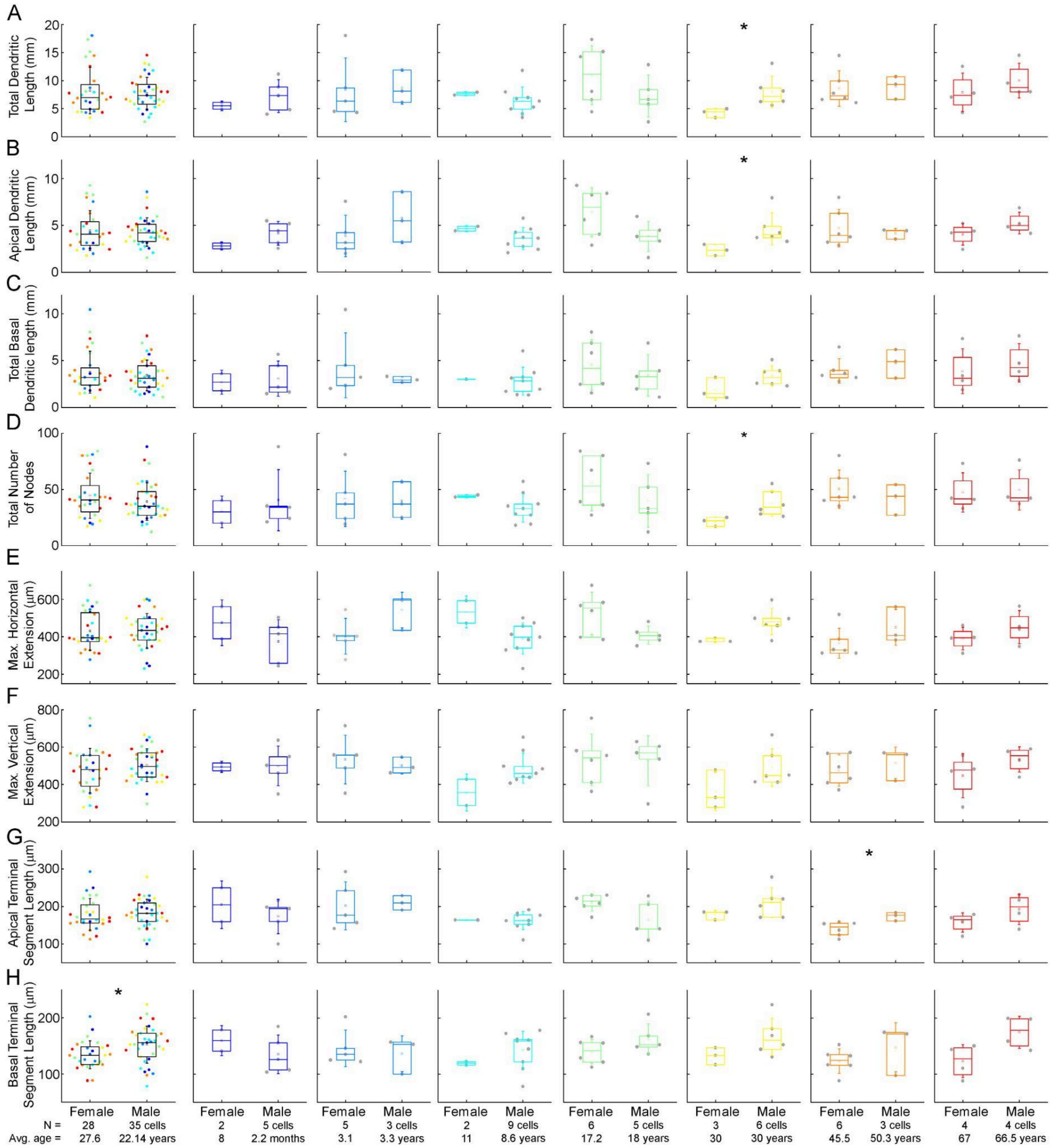

Supplementary Figure 21. Morphological comparison of the examined cells from female and male patients.

A-H: Boxplots showing morphological features: total (A), apical (B), total basal (C), dendritic length, total number of nodes (D), maximal horizontal (E), and vertical (F) extension, average

bioRxiv preprint doi: https://doi.org/10.1101/2024.06.13.598792; this version posted January 6, 2025. The copyright holder for this preprint (which was not certified by peer review) is the author/funder, who has granted bioRxiv a license to display the preprint in perpetuity. It is made available under aCC-BY 4.0 International license.

apical (G) and basal (H) terminal dendritic segment from female and male patients. From left to right: data from all age groups, infant, early childhood, late childhood, adolescence and late adulthood groups.
